# Constant pH Coarse-Grained Molecular Dynamics with Stochastic Charge Neutralization

**DOI:** 10.1021/acs.jpclett.2c00544

**Published:** 2022-04-29

**Authors:** Alexander van Teijlingen, Hamish W. A. Swanson, King Hang Aaron Lau, Tell Tuttle

**Affiliations:** Department of Chemistry, University of Strathclyde, 295 Cathedral Street, Glasgow G1 1XL, United Kingdom

## Abstract

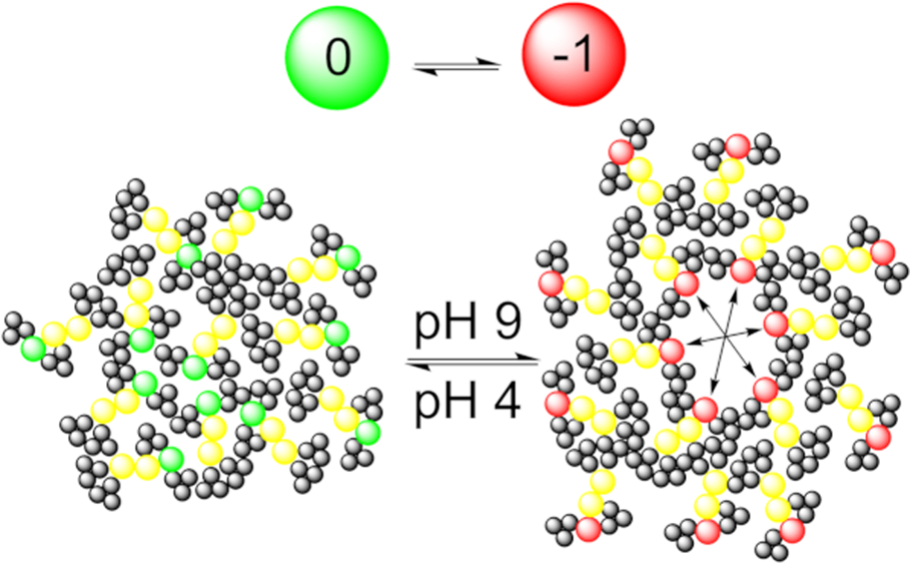

pH dependence abounds in biochemical systems; however, many simulation methods used to investigate these systems do not consider this property. Using a modified version of the hybrid non-equilibrium molecular dynamics (MD)/Monte Carlo algorithm, we include a stochastic charge neutralization method, which is particularly suited to the MARTINI force field and enables artifact-free Ewald summation methods in electrostatic calculations. We demonstrate the efficacy of this method by reproducing pH-dependent self-assembly and self-organization behavior previously reported in experimental literature. In addition, we have carried out experimental oleic acid titrations where we report the results in a more relevant way for the comparison with computational methods than has previously been done.

The time scales of coarse-grained molecular dynamics (CGMD) enable researchers to model the dynamic behavior of large biochemical entities and many smaller molecules interacting at the macroscale. While software exists that readily implements several constraints on the system, such as the number and identity of atoms or beads (*N*), the pressure or volume (*P* or *V*), and temperature (*T*), pH is rarely given consideration, partly due to its conflict with the first constraint where the number of particles are initially and persistently defined. However, pH and by extension p*K*_a_ influence a wide range of research areas include graphene oxide adsorption capacity,^1^ peptide self- and co-assembly,^2^ and protein structure;^3^ even extremophile bacterial ice-binding proteins are hindered at slightly below physiological pH (<6).^4^

Herein, we describe a method of performing constant pH molecular dynamics (CpHMD) with dynamic stochastic charge neutralization on the basis of the method developed by Radak et al.^5^ Modifications have been introduced to enable net charge neutralization as well as for the capture of broad apparent p*K*_a_ shifts in aggregates of minimal low dielectric constants. In the validation of this methodology as well as the exploration of use cases, three self-assembling systems of increasing complexity and with existing experimentally quantified p*K*_a_ changes or pH-dependent phase changes were investigated. The first was the commonplace oleic acid micelle titration, which serves as a general evaluation of the CpHMD methods. However, no quantified Hill coefficient has been reported experimentally; therefore, experiments were carried out, and Hill coefficients were reported. Thus, our method can be accurately evaluated against others, and the experimental data can be used to benchmark future studies. Following this, we perform self-assembly simulations of FmocFF and FFD and find the results from this computational method to be in close agreement with the literature.

The biggest obstacle to overcome in CpHMD is that of the changing atom and bond existence or identity. One method able to bypass this issue is by implementing dummy atoms (Lennard-Jones invisible particles)^6,7^ and switching their charge. Another approach is the use of a reactive force field, which enables one to keep N constant by transferring protons between the solvent and the solute. However, even at pH 4, only a single H_3_O^+^ exists in a 25 nm^3^ box, which implies that unfeasible computational time scales would be required while waiting for the chance encounter of species.

The method we use throughout this work is nonequilibrium molecular dynamics/Monte Carlo (neMD/MC), which benefits from the use of explicit solvent environments while scaling linearly in regard to the number of titratable sites. On top of the original method, we have added stochastic charge neutralization, which seeks to minimize the number of ions in solution while retaining neutral charge. Previous CpHMD methods developed by Chen et al.^8^ and Wallace and Shen^9^ have implemented charge neutralization in continuous pHMD (λ-dynamics) to determine all-atom p*K*_a_ values for six organic molecules and three proteins. These methods reproduced experimental p*K*_a_ using residue–ion/water pairs for charge neutralization, which is more accurate than charge fluctuating methods.^8,9^

We perform grand canonical simulations that are capable of varying the number of particles in a system; however, this was not a necessity in our (NPT) system as with the MARTINI coarse-grained force field only bead type and charge are required to change. In our scheme, conventional CGMD is performed for a number of steps; then, a titratable residue is driven from its current state and its concurrent protonated/deprotonated state (*x*, λ) to a candidate state (*x*′, λ′). The probability of a switch being accepted is taken as the probability of the *x* → *x*′ neMD switch, which is conditional on the λ → λ′ switch and is accepted on the basis of a Monte Carlo Metropolis criterion. λ → λ′ is calculated for each potential change in protonation state and acts as a low-cost estimate of transition probability on the basis of the Hill equation (eq 1, Hill coefficient (*n*) = 1) with the likelihood of deprotonation (*S*) at a given pH evaluated against the Metropolis criteria.
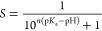
1

Previous CpHMD studies have often attempted to investigate pH-dependent macromolecular effects without conserving charge and allowing the system to fluctuate.^10,11^ There are a number of methods that have been employed to facilitate this, such as using cutoff methods for evaluating electrostatic interactions^10^ or ignoring it (by implicitly neutralizing via Ewald background charge), which is a feasible solution in some cases.^11^ However, this can lead to severe artifacts in systems that lack a uniform dielectric constant (e.g., bilayer, inside a protein, molecular aggregates, etc.).^3,12^ Hub et al. demonstrate and quantify this effect in their study of a dielectrically inhomogeneous system (hexadecane/water) where a particle of charge  is dragged across the hexadecane slab and its free energy profile is measured. It was demonstrated that as background charge becomes increasingly negative the apparent hydrophobicity of the charged particle increases so dramatically that the energy minima of the charged particle is the center of the hexadecane slab.^12^ Ewald methods are generally more accurate in evaluating electrostatic interactions, which are by their nature long-range interactions, than cutoff based methods, which are liable to produce artifacts.^13,14^ Therefore, we use particle-mesh Ewald (PME)^15^ summation techniques to calculate electrostatic interactions in all of our systems, unless explicitly comparing electrostatic evaluation methods.

Simulations of each peptide were set up using the NAMD^16^ and VMD^17^ software packages; these programs were also used to measure solvent accessible surface area (SASA). Visualizations of molecular macrostructures were rendered by the software package OVITO.^18^ SASA was used to determine the aggregation propensity (AP; eq 2) score by measuring this value at the beginning and various points throughout the MD simulations.^19,20^
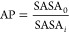
2

The parameters for coarse-grained amino acids, water, and ion molecules are those of the MARTINI force field (version 2.1).^21^ The amino acid atoms are mapped ca. one-to-four in corresponding heavy atoms-to-beads; the water beads represent four water molecules following the same mapping ratio for computational efficiency. Due to the relationship between the diffusion constants of the MARTINI coarse-grained and atomistic simulations, the effective simulation time is 4 times greater than the formal simulation time. Herein, we refer to the effective simulation time and not the formal time. Computational methods for each system investigated can be found in Supporting Information Sections 1.1–1.3.

Due to the aforementioned artifacts that can arise from Ewald implicit background charge, we develop a method for dynamic charge neutralization, which is particularly suited to coarse-grained systems. The constant charge method implemented herein leverages the coarse-grained nature of the MARTINI force field by stochastically altering water bead charges after each cycle according to eq 3. Without abruptly adding any beads or bonds or changing coordinates, the mapping of MARTINI beads is such that the positive form resembles an Eigen cation (H_9_O_4_^+^).^22^ This required some minor changes to the NAMD source code, which have been made available on GitHub.
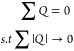
3

To evaluate our constant charge method, we initially measure computational and experimental Hill coefficient p*K*_a_ shifts for oleic acid micelles as a means to test and benchmark this method against other methods of investigating pH effects on this molecule’s macrostructure.^7,10,23−25^ We demonstrate the differences using PME vs a switched cutoff for oleic acid micelles and oleic acid in a phospholipid bilayer (PLB) as well as fluctuating vs constant charge (CC). Following this, we demonstrate that our model, using PME+CC, can reproduce experimentally observed pH-dependent phenomena in FmocFF and FFD self-assembled systems, which for the former we have developed a generally applicable Fmoc MARTINI coarse-grained model (Supporting Information Section 1.2.1).

A survey of the literature produces a wide range of results for fatty acid (FA) p*K*_a_ and Hill coefficient shifts owing in part to the use of different CpHMD methods and limited experimental data (Table 1); however, consistent features that emerge are

- Apparent p*K*_a_ increases when FAs form micelles and the p*K*_a_ shift is proportional to the size of the micelle
- Titration of FA micelles is anticooperative
- Apparent p*K*_a_ shifts of FAs are greater in phospholipid bilayers than in self-assembled micelles
- Titration of FAs in phospholipid bilayers is less anticooperative than in self-assembled micelles due to the stabilizing effect of charged and polar head groups
- Ions stabilize deprotonated FAs and reduce anticooperativity^11,23^

**Table 1:** Measured p*K*_a_ and Hill Coefficient Values of Oleic Acid in Water, Micelles, and Phosphatidylcholine Bilayers^a^

		electro.	charge	environment	p*K*_a_	Hill coef.
A1	this study	PME	constant	water	4.73 ± 0.03	1.0 ± 0.05
A2	this study	switch	fluctuating	30-mer	5.20 ± 0.02	0.86 ± 0.03
A3	this study	switch	constant	30-mer	5.18 ± 0.02	0.82 ± 0.02
A4	this study	PME	fluctuating	30-mer	7.38 ± 0.05	1.0 ± 0.11
A5	this study	PME	constant	30-mer	6.92 ± 0.05	0.89 ± 0.09
A6	this study	PME	constant	POPC	5.27 ± 0.03	1.0 ± 0.07
B1	this study	experimental^b^		1.0 M	6.55 ± 0.01	0.86 ± 0.01
B2	this study	experimental^b^		2.0 M	6.55 ± 0.01	0.85 ± 0.01
B3	this study	experimental^b^		5.0 M	6.73 ± 0.02	0.86 ± 0.02
B4	this study	experimental^b^		10.0 M	7.33 ± 0.03	0.65 ± 0.03
C1	Bennett et al.^10^	switch	fluctuating	20-mer	6.3	0.51
C2	Bennett et al.^10^	switch	fluctuating	30-mer	6.5	0.49
C3	Bennett et al.^10^	switch	fluctuating	DOPC	6.6	0.96
D1	Grünewald et al.^7^	PME	constant	water	4.62	
D2	Grünewald et al.^7^	PME	constant	POPC	5.29	

a CpHMD results are time and molecule averaged. Reported p*K*_a_ and Hill coefficients have ranged from 4.6 to 9.85 and 0.40 to 1.0, respectively, depending on the environment.^11,26−29^ This table lists only previous studies performed in similar conditions to this one.

b Experimental details and data available are in Supporting Information Sections 1.1.2 and 2.1.

CpHMD simulations of aggregating oleic acid molecules (Figure 1) are in accordance with the first point as the degree of aggregation (AP) decreases the degree of deprotonation decreases, thus shifting the p*K*_a_.

**Figure 1:**
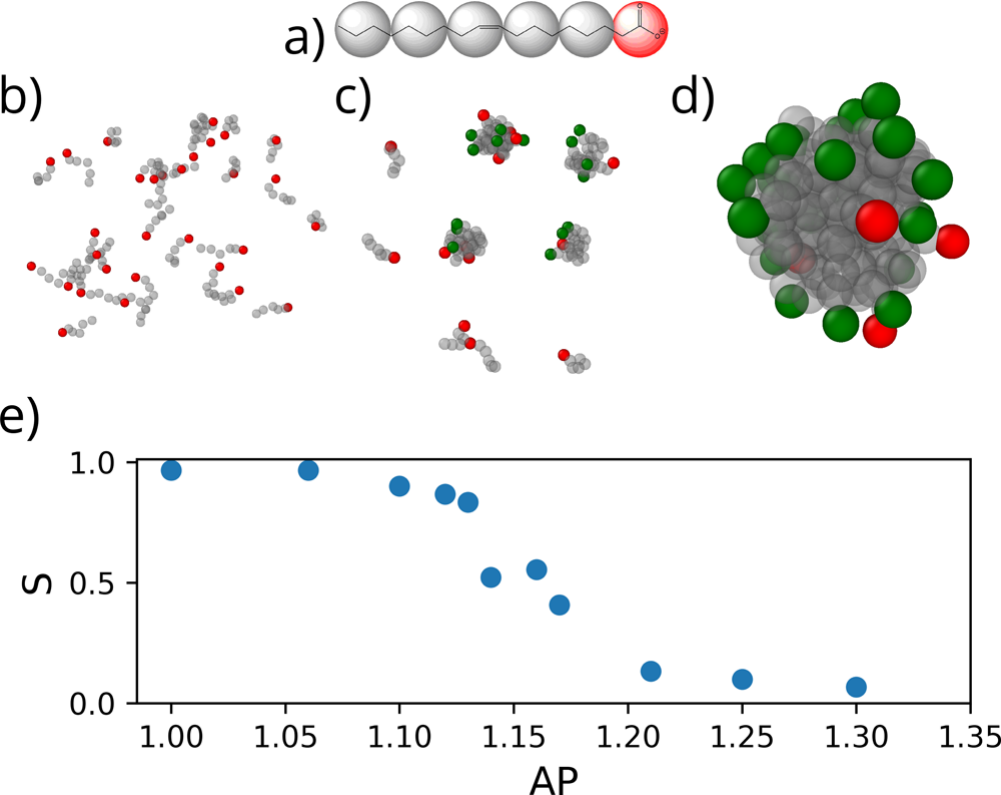
(a) Oleic acid overlaid with coarse-grained structure. (b–d) Increasing p*K*_a_ due to micelle formation at pH 6 is shown by the ratio of red (deprotonated) to green (protonated) oleic acid molecules. In monomeric form (b), all molecules are deprotonated; as they begin micelle formation, (c) more become deprotonated leading to (d) 30-mer micelles at which deprotonation drops to ∼50%. (e) Relative deprotonation vs average AP of oleic acid molecules at that degree of deprotonation.

In order to investigate the p*K*_a_ and Hill coefficient changes in oleic acid micelles using different methodologies, we equilibrate a 30-mer fully deprotonated micelle and then perform CpHMD at pH 4 and 5, followed by 0.5 increments up to pH 8. The p*K*_a_ and Hill coefficient were fit from the average deprotonation of the first continuous 100 ns of each simulation where the standard deviation of the mean protonation state of the micelle is less than 0.05 for PME and 0.1 for non-PME. This allows the initial usually poor guess at protonation states to be excluded from the analysis (Supporting Information Section 1.1). We find that using PME electrostatics shifts the p*K*_a_ more so than the switched cutoff (Table 1, A2–A5). We also find PME produces a wider gap between p*K*_a_ and cooperativity values for fluctuating vs constant charge due to the background charge implicit in the Ewald algorithm and the stabilizing effect of counterions, respectively (Table 1, A2–A5). This is to be expected as more headgroup–headgroup interactions are captured via PME electrostatics. We also find that phospholipid bilayers shift the p*K*_a_ to higher values without as large an associated Hill coefficient shift due to the stabilizing effect of the phospholipid head groups on the charged oleate headgroup (Table 1, A6).

Previous simulation studies have tended to produce titration curves with overly depressed Hill coefficients. While experimental FA titrations do not usually report any Hill coefficient values, the visual inspection of published titration curves indicate they are ∼0.8–0.9.^26−29^ For oleic acid titration performed for the purposes of this study, we have explicitly reported fitted Hill coefficients (Table 1, B1–B4) to effectively compare these methods as well as provide an effective experimental benchmark for future CpHMD studies (for experimental details, see Supporting Information Sections 1.1.2 and 2.1). We find our algorithm with PME+CC as well as the work of Bennett et al.^10^ to accurately reproduce the p*K*_a_ though our results show better reproduction of the Hill coefficients (Table 1, A5, B1–B3, C3). This is due to the difference in algorithm used where the simulation methodology of Bennett et al.^10^ allows the degree of deprotonation to exist continuously along a 7-point spline function (Table 1, C1–C3); the method described herein produces a binary state for each molecule, which better allows for fully (de)protonated states for entire micelles at high and low pHs, which produces a Hill coefficient closer to 1.

Next, we investigate the relationship reported by Tang et al. between FmocFF concentration and apparent p*K*_a_ shifts;^30^ using CpHMD, we are able to find both of these shifts (Table 2) within the margin of error (<1 p*K*_a_ unit). p*K*_a1_ is observed within an aggregate of 600 FmocFF molecules representing a large aggregate formed in a strongly basic condition (fully deprotonated). In order to better capture this large shift, the cheap preswitch function (λ → λ′) was disabled and a full switch trajectory was run at every iteration. The number of steps for performing the test switch was also increased from 200 to 1500 to increase the precision, as this is now the only determining factor in performing a switch (other than MC acceptance). The relatively lower apparent p*K*_a_ (p*K*_a2_) reported is seen in aggregates from 10 to 600 molecules.

**Table 2:**
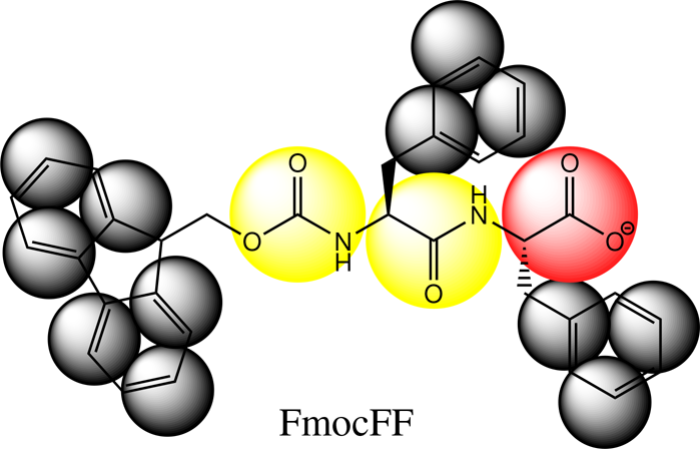
p*K*_a_ Shifts of Our Method and Tang et al.^30^^,^^a^

	our method (comp.)			Tang et al.^30^ (exp.)	
	p*K*_a_	Hill coef.	conc. (mmol/L)^b^	p*K*_a_	conc. (mmol/L)^b^
p*K*_a1_	9.68	0.47	348	9.5–10.2	≥5
p*K*_a2_	4.48–5.0	0.84–1.0	8.5–348	5.2–6.2	≥1
p*K*_a_	3.61	1.0	0.85	3.5	≥0.01

a FmocFF overlaid with a coarse-grained structure. Two p*K*_a_ shifts can be observed for FmocFF aggregates: p*K*_a1_ exists when titrating from a high pH at a high concentration where many molecules become embedded deep within the aggregate. p*K*_a2_ is found in all aggregates of FmocFF, which is induced by interacting surface N-termini.

b Concentrations in molecular simulations are necessarily much higher than the equivalent experimental method in order achieve feasible computation time and to capture macro-structural effects in the nanoscale simulation box.

We rationalize that the higher apparent p*K*_a_ (p*K*_a1_) is produced by the different dielectric constant between the surface of the aggregate exposed to water and the monomers buried deep within the aggregate that experience a much lower dielectric environment. The lower apparent p*K*_a_ (p*K*_a2_) is due to interacting head groups that proliferate at the aggregate surface at moderate concentration (visualized in Figure S5). From the simulations, it is evident that as the nanoaggregate size increases the manoeuvrability of head groups increase, which in part causes the observed reorganization of molecules (*spherical* → *oblong* → *fibrous*) to maximize headgroup distance (Figures S6–S11). This has the effect of decreasing the apparent p*K*_a_ and increasing apparent cooperativity due to fewer charge–charge interactions (Figure 2).

**Figure 2:**
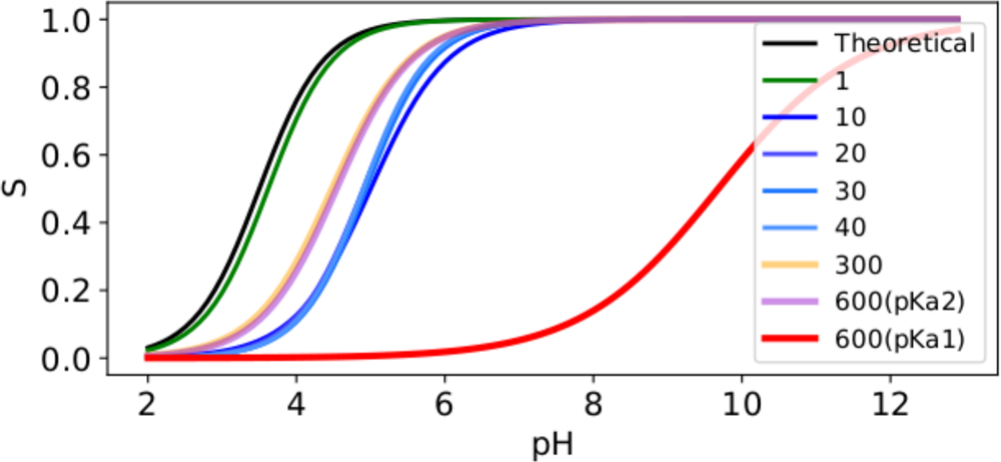
With only one molecule (green), the titration closely matches the theoretical p*K*_a_ (black); the apparent p*K*_a_ increases by ∼2 units for small spherical aggregates (10–40 molecules, blue) but only by ∼1 for the surface interactions of the larger aggregates (orange and purple), while for the hydrophobic core of the largest aggregate (red) the p*K*_a_ is up to 6 p*K*_a_ units greater than the theoretical p*K*_a_.

Due to the highly hydrophobic nature of FmocFF and the large degrees by which the apparent p*K*_a_ of FmocFF can shift, hydrogels of this Fmoc-dipeptide are not stable at low pH. To investigate this effect, we constructed a 9 nm long FmocFF tube with an outer radius of 4.5 nm and internal radius of ca. 2.2 nm using the GROMACS *insert-molecules* program with a list of trial cylindrical points. This structure was simulated in water for 4.2 μs using the two-step switch evaluation approach. Both pHs initially experience a rapid reorganization while retaining the nanotube structure. At pH 4, the FmocFF molecules gradually undergo syneresis as the nanotube begins to collapse, creating a less polar internal environment, which promotes further collapse; this does not occur at pH 9 where the Phe residues remain deprotonated and the nanotube structure is stable (Figure 3). This is in agreement with the experimental results published by Tang et al.^30^ as well as the experimental survey of Fmoc-dipeptide reported by Adams et al., which found that, at an approximate range of logP 2.8–5.5 (Fmoc-AV/VG/LA/FA/LG/FG/FV/LF), Fmoc-dipeptides formed hydrogels with an acid pH trigger; however, above this range, FmocFF was found to be too hydrophobic and only form a gel under basic conditions.^31^

**Figure 3:**
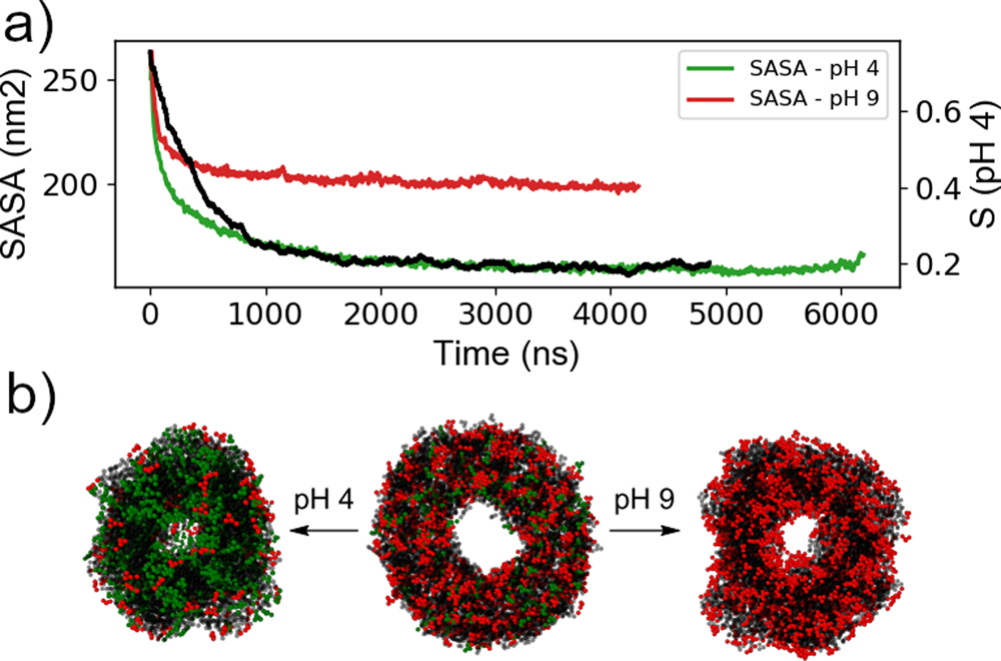
(a) SASA over time for pH 4 and 9 showing syneresis at pH 4 and stability at pH 9 as well as the correlation between SASA and mean deprotonation at pH 4. (b) The initial nanotube structure (middle) and the collapsed aggregate at pH 4 (left) and stable nanotube at pH 9 (right). Green and red beads represent protonated and deprotonated Phe residues.

The pH-dependent structural effects of another peptide, FFD (Figure 4a), have previously been reported both computationally (fluctuating pH) and experimentally to self-assemble into bilayers and hydrogels at a pH between 5 and 7.5.^2,32,33^ We extend our investigation of long-range macromolecular self-assembly to pH 2–10 and find that at pH < 3.0 large unstructured aggregates form; at 3.0 ≤ pH < 9.0, bilayer structures dominate while at pH ≥ 9.0 bilayer structures thin out to form nanowires due to the rearrangement of unstabilized negative charges to the edges (Figure 4b).^23^

**Figure 4:**
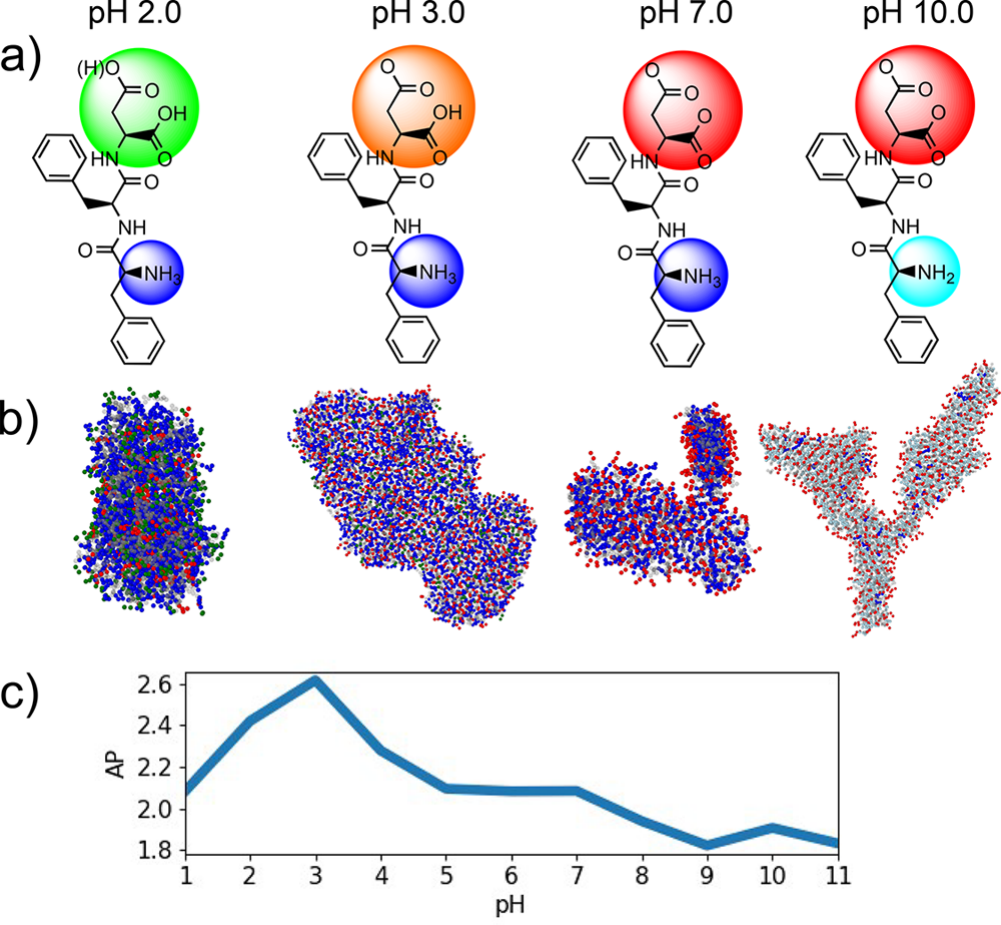
(a) Skeletal structures with mean charge overlays of different groups: carboxylic acid (green), carboxylate (red), amine (turquoise), ammonium (blue); intermediate colors are intermediate charge states at different pHs. (b) Snapshots of major molecular aggregates with titratable beads colored as previously described. Complete and larger scale FFD macrostructures snapshots are available in Figures S12–S22. (c) AP is high for large unstructured agglomerates (pH 1 and 2) and long-range bilayers (pH 3–7) but lower for nanowire structures (pH ≥ 9).

FFD is reported to have an AP of 2.14 when simulated with reaction-field electrostatics without constant pH and all titratable groups charged (which is the approximate charge state found at pH 7).^33^ Our results are in line with both sets of observations, having an AP of 2.08 at pH 7.0 with both carboxylate groups deprotonated and the N-terminus group protonated >95% of the time. The highest degree of aggregation at pH 3 (AP = 2.62) is due to having the most favorable mean charge states for forming bilayers (charge = −0.22, Σ|charge| = 2.22). This creates strong hydrophilic interactions on the surface while minimizing intermolecular repulsion. At lower pH, the surface is less hydrophilic and too self-repulsive (pH 2 charge = +0.56, Σ|charge| = 1.16) to form bilayers, and at high pH, the high net charge impedes bilayer formation (AP = 1.91, charge = −2.0), favoring thin nanowires with a larger surface area to disperse negative charges.

In conclusion, a CpHMD method has been extended to include a novel function for conserving net zero charge. This method has been used to simulate three self-assembling coarse-grained systems. The factors that influence p*K*_a_ shifts and how pH modulates macromolecular structure have been elucidated from these examples. In the method developed here, we are able to turn on/off charges without massive disruption to the system in order to maintain a net neutral charge; in turn, this allows for the use of PME in a manner that does not induce a background charge that propagates to unreasonable regions of the system. The method we have developed leverages the benefits of Ewald electrostatic techniques, constant charge, and coarse-graining, which both enables one to access the longer time scales required to observe self-assembly and is in agreement with existing computational and available experimental findings. Many systems currently under investigation could benefit from a constant pH approach even at pH 7 as differences in the local environments can shift p*K*_a_ values above and below 7, making the assumed charge states potentially inaccurate without constant pH MD.

## Supporting Information Available

Computational procedures; additional snapshots of aggregated peptides (PDF)
Transparent Peer Review report available (PDF)

## Notes

All data underpinning this publication are openly available from the University of Strathclyde KnowledgeBase at 10.15129/f76ac245-0e38-40ca-ac3c-26c000510faf.
