# Supplementary material for: Constant pH Coarse-Grained Molecular Dynamics with Stochastic Charge Neutralization
Source: J Phys Chem Lett. 2022 Apr 29;13(18):4046–51. doi: 10.1021/acs.jpclett.2c00544 (PMC9109222; doi:10.1021/acs.jpclett.2c00544)
Supplement: Supplementary file 1 — jz2c00544_si_001.pdf [file jz2c00544_si_001.pdf]

Constant pH Coarse-Grained Molecular Dynamics with Stochastic Charge Neutralization

Alexander van Teijlingen, Hamish W. A. Swanson, King Hang Aaron Lau, Tell Tuttle*

Department of Chemistry, University of Strathclyde, 295 Cathedral Street, Glasgow, G1 1XL, UK

Email: tell.tuttle@strath.ac.uk

Phone: +44 141 548 2290

Table of Contents

1. Experimental Procedures 3
1.1 Oleic acid 3
1.1.2 Oleic acid lab titration 3
1.2 FmocFF 3
1.2.1 MARTINI Fmoc model 5
1.3 FFD 5
2. Results 6
2.1 Oleic acid lab titration 6
2.2 FmocFF Frames 7
2.2 FFD Frames 11
3. File Archive 22
4 References 23

Supporting Information

1. Experimental Procedures

All simulations were NPT, performed using Langevin dynamics with a target temperature of 298 K and isotropic (except for the oleic acid + PC bilayer system which was semiisotropic) pressure coupling using a modified Nosé-Hoover method were Langevin dynamics is used to control fluctuations in the barostat, with a target of 1.01325 bar, a period of 2 ps and decay of 1 ps. The pair list distance was 15 Å and short range non-bonded interactions as well as full electrostatic evaluation calculated at every timestep. PME grid spacing was set to 1.2 Å, where switched electrostatics were used the switching distance was 9 Å with the cut-off at 12 Å.

1.1 Oleic acid

30 oleic acid molecules were inserted into a 20 nm^3 cubic water box and equilibrated using a Berendsen barostat until a single micelle had formed. Using a timestep of 25 fs the system was minimized for 2,000 steps and CpHMD iterations of 200 steps were performed with 200 steps for the switch trajectory were run until the moving standard deviation of mean deprotonation was < 0.05 for PME electrostatics and < 0.1 for non-PME electrostatics. In the case of using only 1 oleic acid molecule (single molecule in water, single molecule in DOPC bilayer) we take the first 100 ns as standard deviation is not a valid option for a single binary measurement.

1.1.2 Oleic acid lab titration

Oleic acid was dissolved in 0.1 M NaOH (reagent grade, Sigma Aldrich) at each concentration of interest (1, 2, 5 and 10 mmol/L). The volume of solution was 8 mL. A Jenway pH Meter (model 3510) was calibrated before use and was used to make all subsequent measurements. A starting pH of ~12.8 was reached once the oleic acid solution was fully dissolved and equilibrated with constant stirring, and then 0.1 M HCl was added until pH 2 was reached. Initially larger volumes were added by burette (e.g., 0.5 mL), however as the gradient began to significantly change, additions were made by pipette (80 to 20 µL) to generate a density of data points at the relevant inflexion points. In plotting the data an adjustment was made for the initial neutralisation of NaOH by dissolution of oleic acid.

1.2 FmocFF

1 - 300 FmocFF molecules were inserted into a 12.5 nm^3 (15 nm^3 for tube structure) cubic water box along. Using a timestep of 15 fs the system was minimized for 2,000 steps up to 50,000 CpHMD iterations of 1000 steps were performed with 200 steps for the switch trajectory. Simulations were stopped early if mean deprotonation had converged.

In the case of the preformed 600 FmocFF aggregate, 600 FmocFF molecules were inserted into a 14.2 nm^3 water box, minimized and equilibrated for 400 ns to form the pseudo-high pH aggregate. CpHMD was run from the final frame with a timestep of 15 fs for between 500 (pH 1) and 87,000 (pH 5) iterations of 500 steps with 1,500 steps for the switch trajectory, until the mean deprotonation had converged, this occurs rapidly at pH 1 and gradually around the pKa values (Figure S1). All values were calculated from the final 10 % of output values (Figure S2) of these simulations, thus excluding iterations where the system was far from convergence on deprotonation states. In this case the

initial protonation states of the monomers are irrelevant to the result as the aggregate is pre-formed.

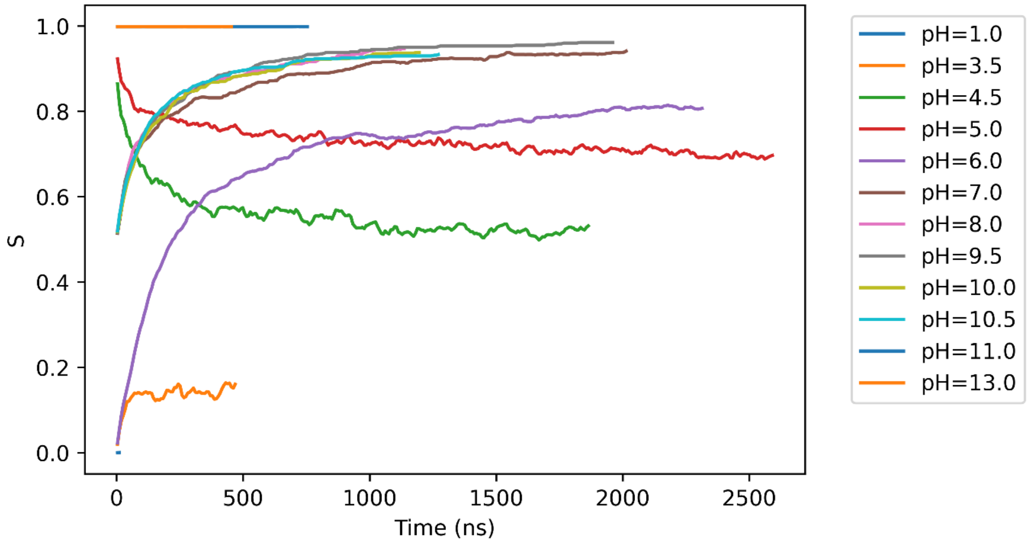

Figure S1. Mean deprotonation (S) of FmocFF molecules over the course of simulation. Simulations were terminated once values converged.

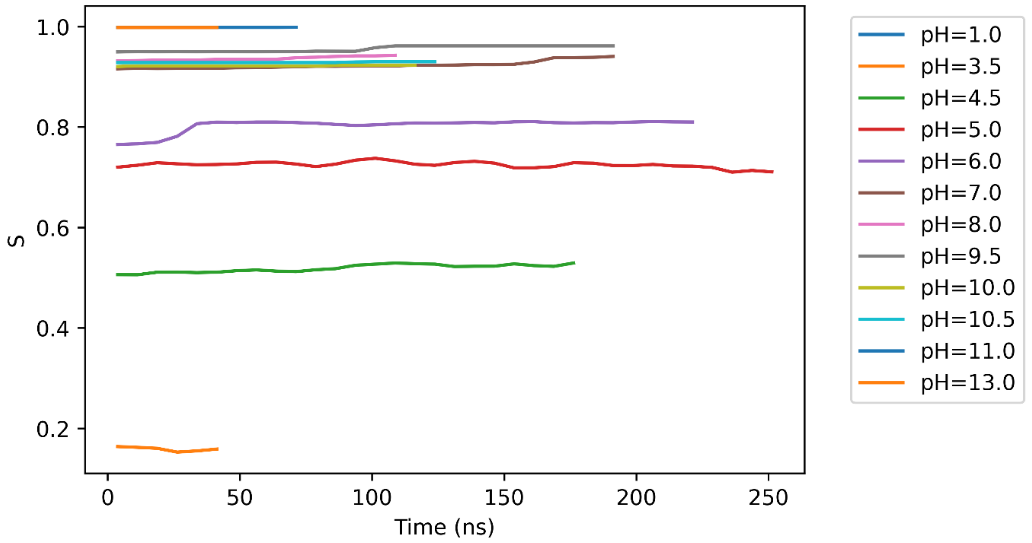

Figure S2. Mean deprotonation (S) of FmocFF molecules during the final 10 % of simulation time, all values have converged.

1.2.1 MARTINI Fmoc model

The MARTINI Fmoc protecting group was produced according to the mapping scheme provided (Figure S3). Bead types were selected to ensure a close relation to the model for phenylalanine in the MARTINI 2.1 forcefield, accordingly a three to one mapping scheme was used with SC4 beads (mass 45 amu). To model the ester linkage a P5 bead was used (mass 72 amu). It is in this same manner that bonded terms were chosen. Improper dihedrals were defined between ring beads to ensure planarity and the equilibrium bond length between the two benzene rings were inequivalent (e.g., bond length at back of the ring was longer than at the front, which connects to the P5 beads) to produce a more triangular shape within the planar system. Atomistic data was generated using the Fmoc model produced by Tuttle *et al*.[1] To validate the model, as well as reproducing the experimental findings of Adams *et al*. in this study, the assembly of two Fmoc based short peptides[2,3] were produced using the model. These findings will be reported at a later date along with the model for general use within the MARTINI framework.

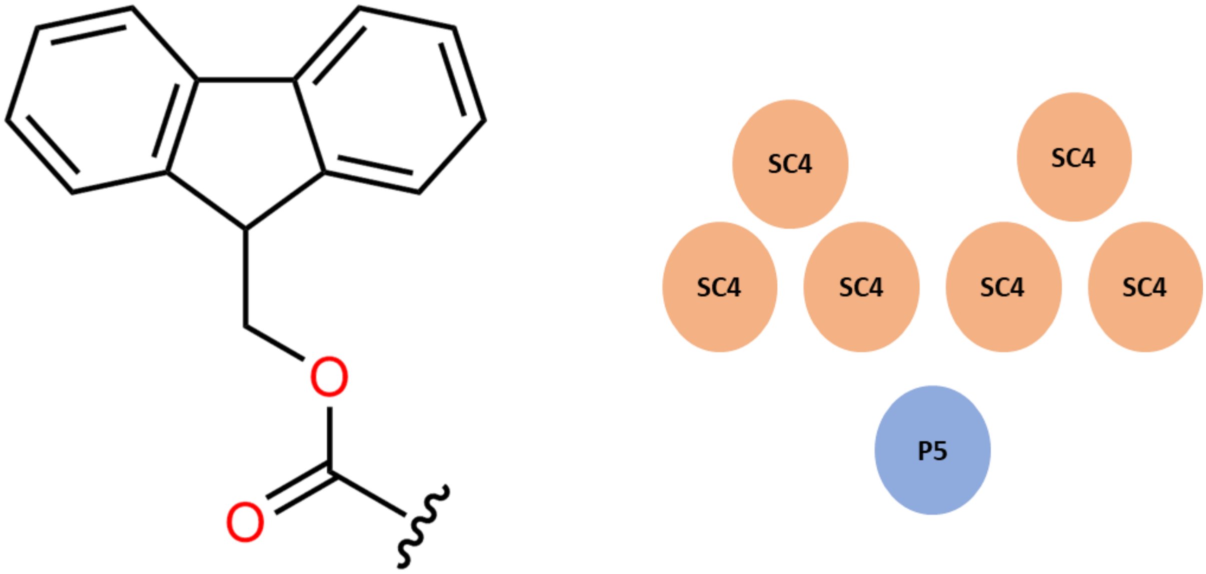

Figure S3. Atomistic representation of the Fmoc protecting group (left) and its MARTINI coarse grain bead representation with bead types shown (right).

1.3 FFD

1,200 FFD tripeptides molecules were inserted with an exclusion radius of 0.3 nm into a 20 nm^3 box along with 1,200 counterions, minimized for 2,000 steps and 30,000 CpHMD iterations of 1000 steps were performed with 500 steps for the switch trajectory both using a timestep of 15 fs (1800 ns effective MARTINI simulation time).

2. Results

2.1 Oleic acid lab titration

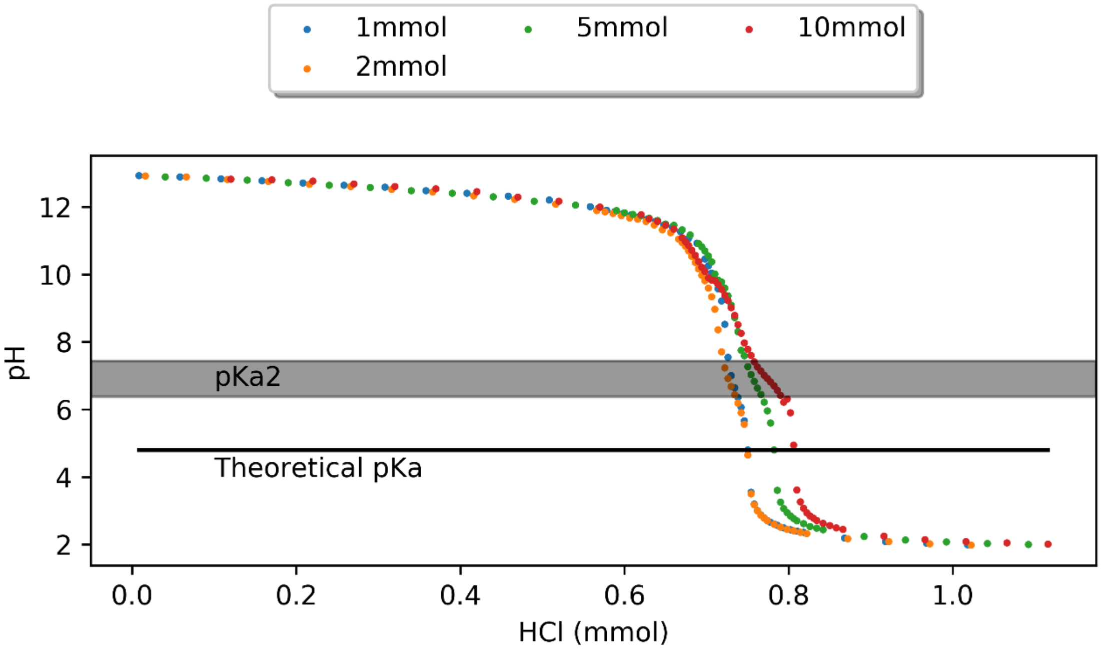

Figure S4. Titration graph of oleic acid at four different concentrations, each showing a shifted pKa at between pH 6.4 and 7.4.

2.2 FmocFF Frames

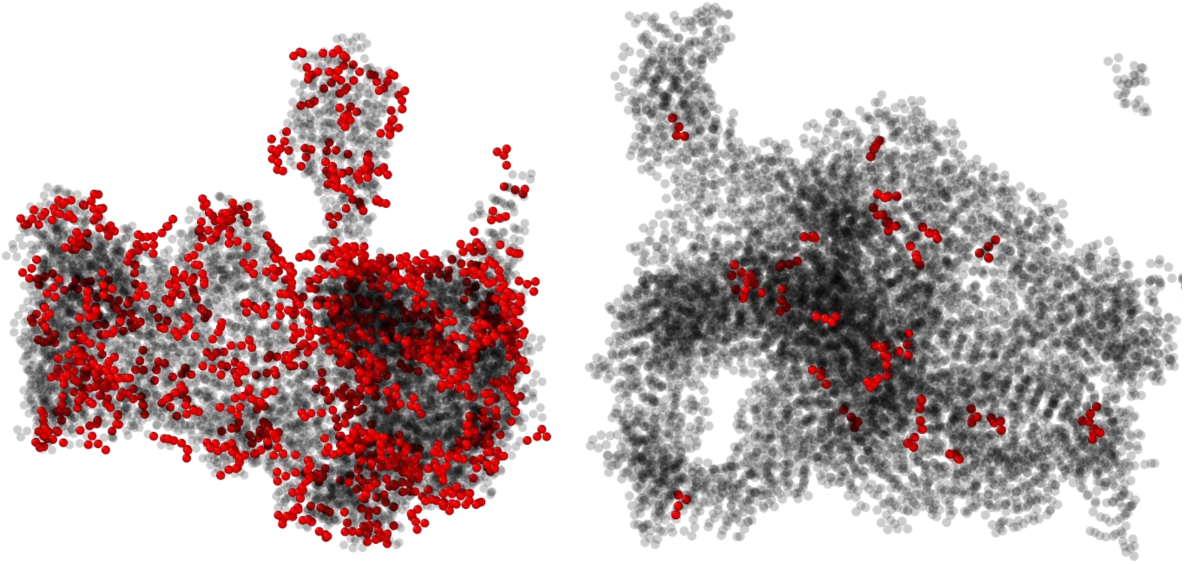

Figure S5. 600 FmocFF molecules preformed at high pH, red beads represent deprotonated residues with pKa ~ 5.3 (Left) and pKa ~9.5 (Right), semi-transparent black beads represent all other beads.

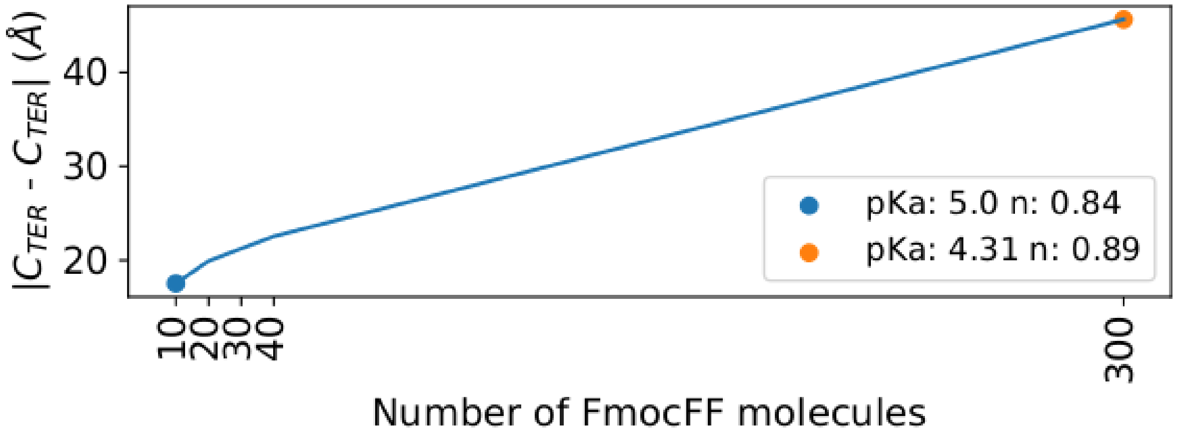

Figure S6. Mean charged residue distance (at pH 5) increases with the number of FmocFF molecules.

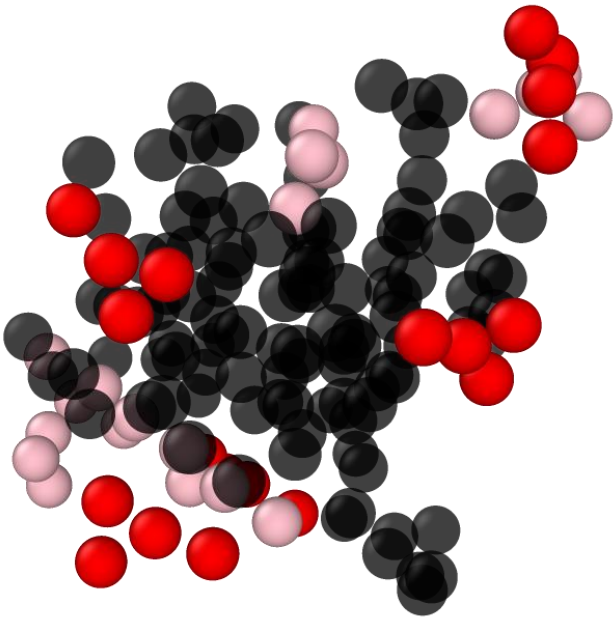

Figure S7. 10 FmocFF molecules at pH = 5.0, red beads represent deprotonated residues, pink beads represent protonated beads while semi-transparent black beads represent other F and Fmoc residues.

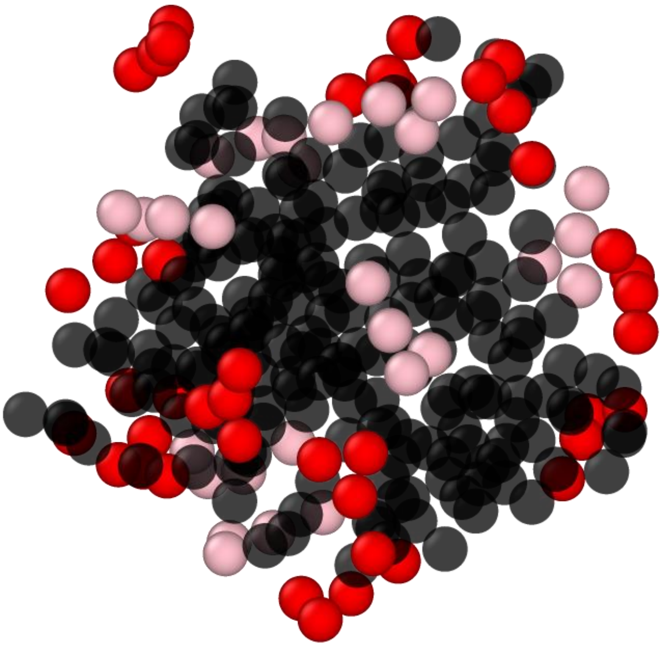

Figure S8. 20 FmocFF molecules at pH = 5.0, red beads represent deprotonated residues, pink beads represent protonated beads while semi-transparent black beads represent other F and Fmoc residues.

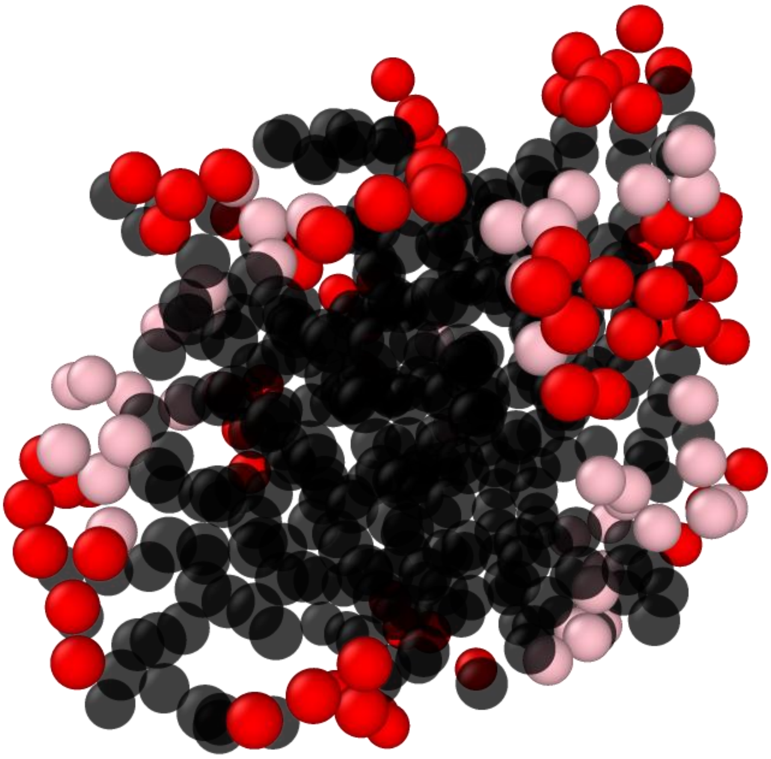

Figure S9. 30 FmocFF molecules at pH = 5.0, red beads represent deprotonated residues, pink beads represent protonated beads while semi-transparent black beads represent other F and Fmoc residues.

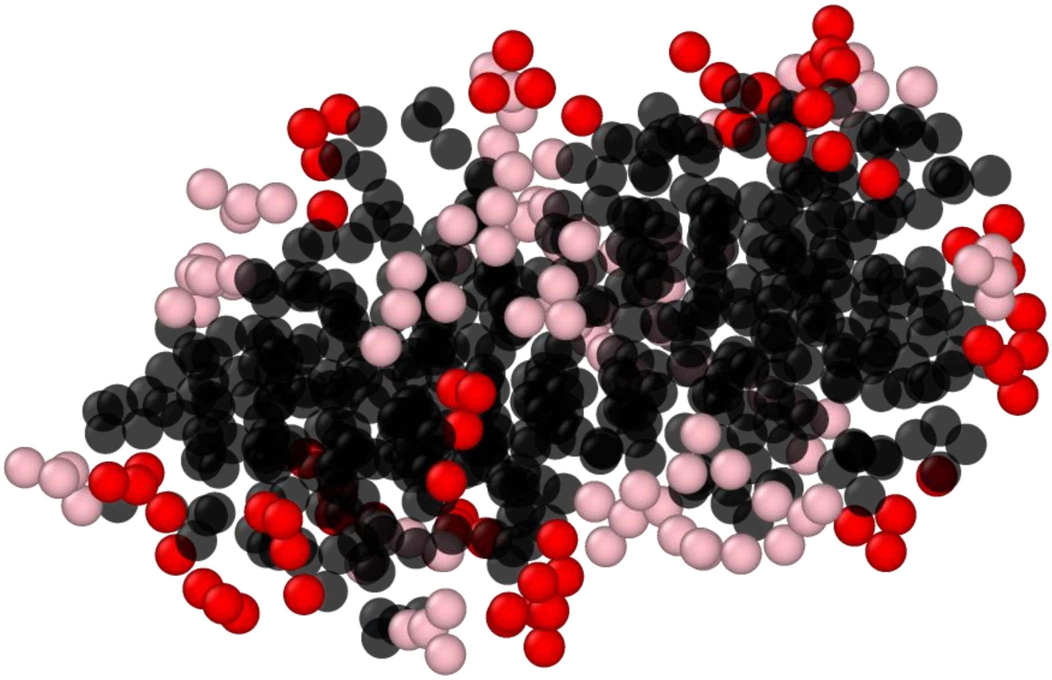

Figure S10. 40 FmocFF molecules at pH = 5.0, red beads represent deprotonated residues, pink beads represent protonated beads while semi-transparent black beads represent other F and Fmoc residues.

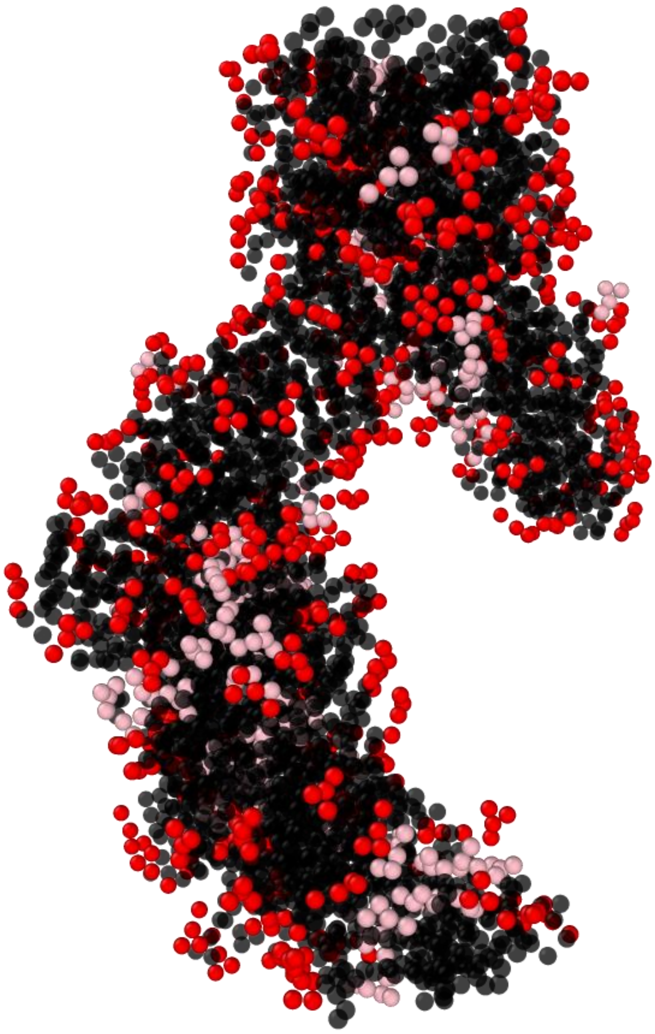

Figure S11. 300 FmocFF molecules at pH = 5.0, red beads represent deprotonated residues, pink beads represent protonated beads while semi-transparent black beads represent other F and Fmoc residues.

2.2 FFD Frames

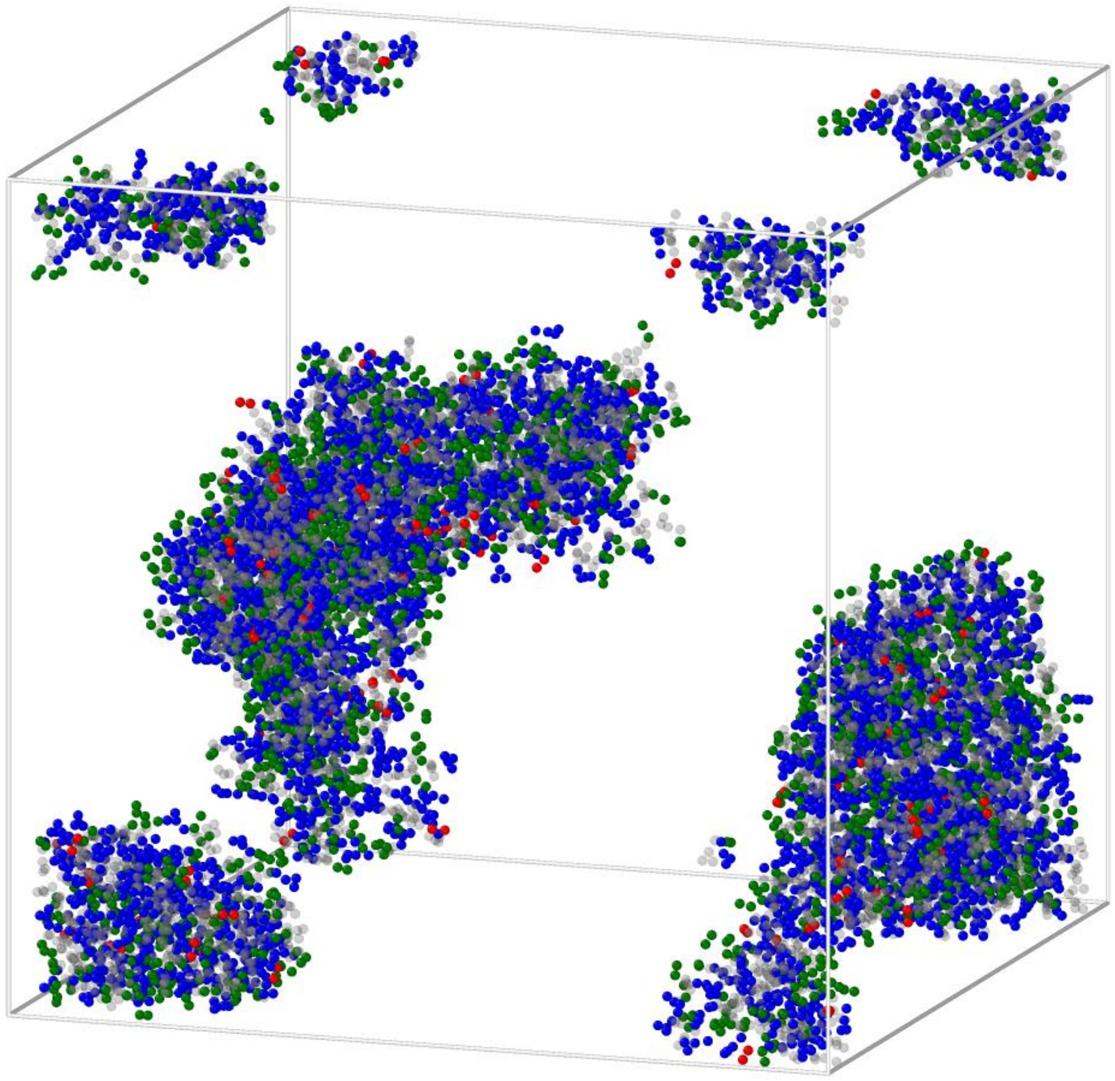

Figure S12. 1200 FFD molecules at pH 1, dark blue beads show protonated F residues (NH_3^+), red represents deprotonated carboxyl residues (COO^-) pink represents protonated carboxyl residues (COOH) while grey represents position 2 F residues. At this pH deprotonated F residues dominate leading to a high net positive charge and the inability to form bilayers.

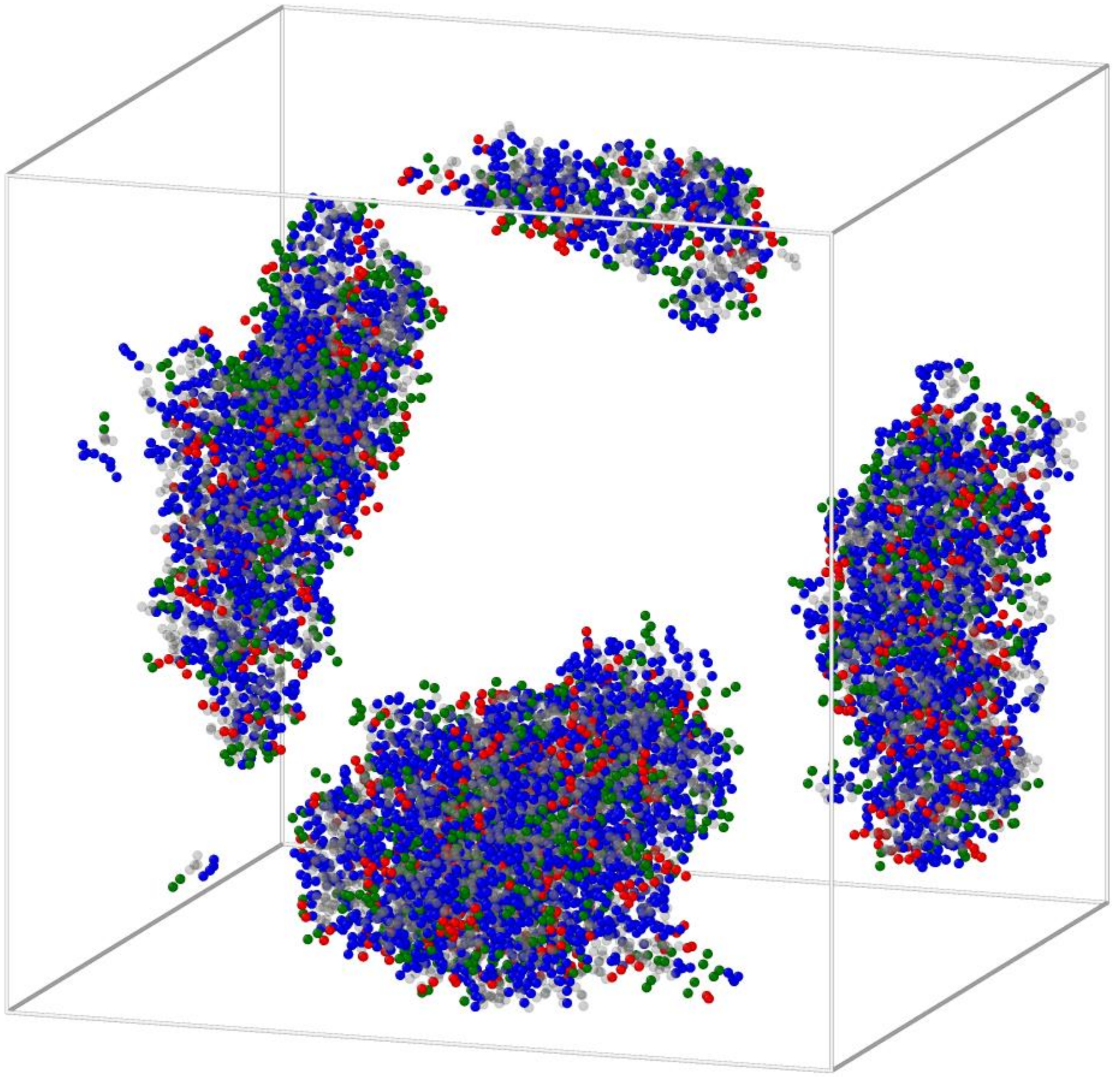

Figure S13. 1200 FFD molecules at pH 2, dark blue beads show protonated F residues (NH_3^+), red represents deprotonated carboxyl residues (COO^-) pink represents protonated carboxyl residues (COOH) while grey represents position 2 F residues. At this pH deprotonated F residues dominate leading to a high net positive charge and the inability to form bilayers.

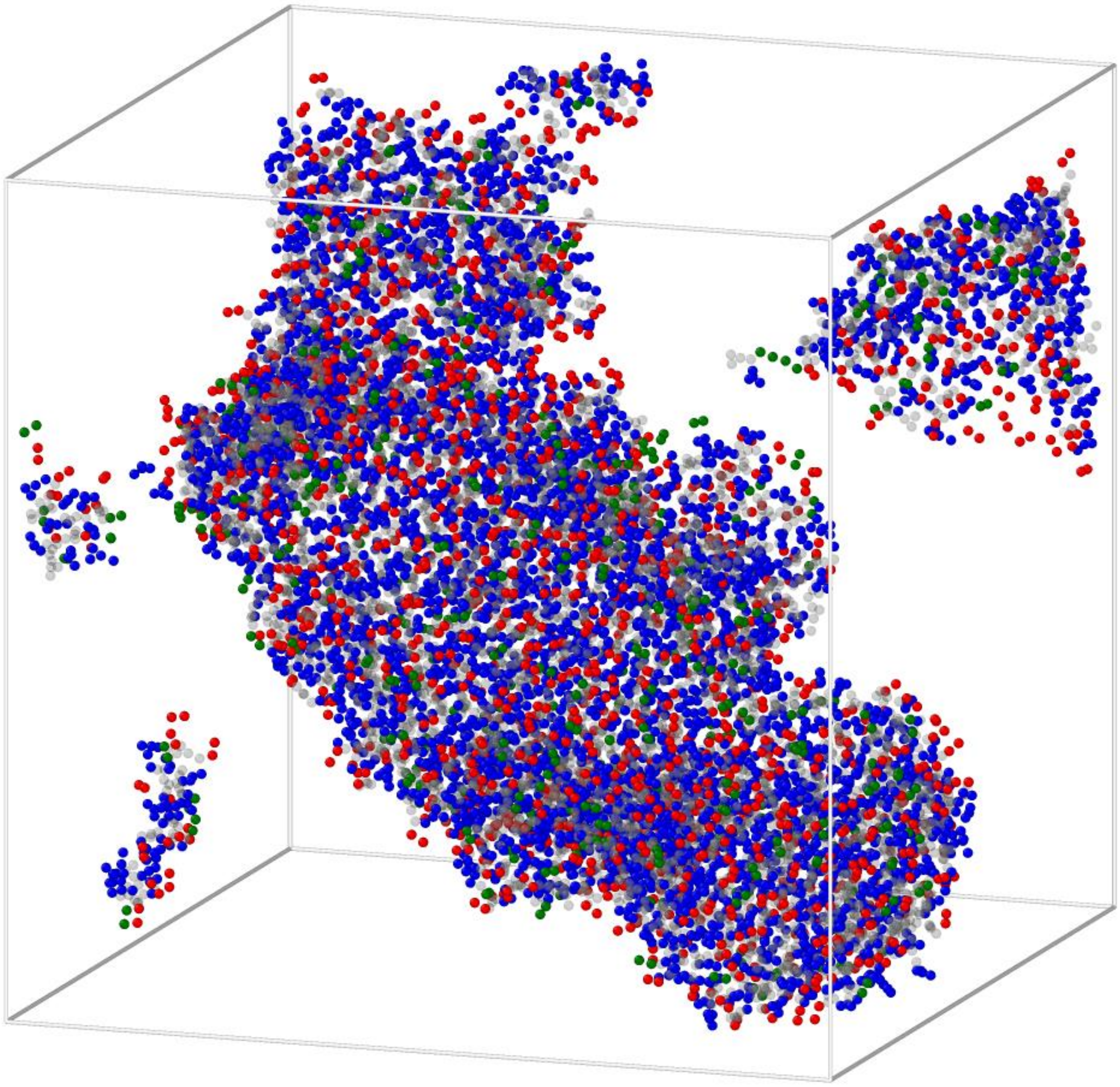

Figure S14. 1200 FFD molecules at pH 3, dark blue beads show protonated F residues (NH3+), red represents deprotonated carboxyl residues (COO-) pink represents protonated carboxyl residues (COOH) while grey represents position 2 F residues. At this pH charge groups are nearly even leading to a near zero net charge and the ability for bilayers to form due to high hydrophilicity paired with charge-charge attraction to stabilize the charged surface.

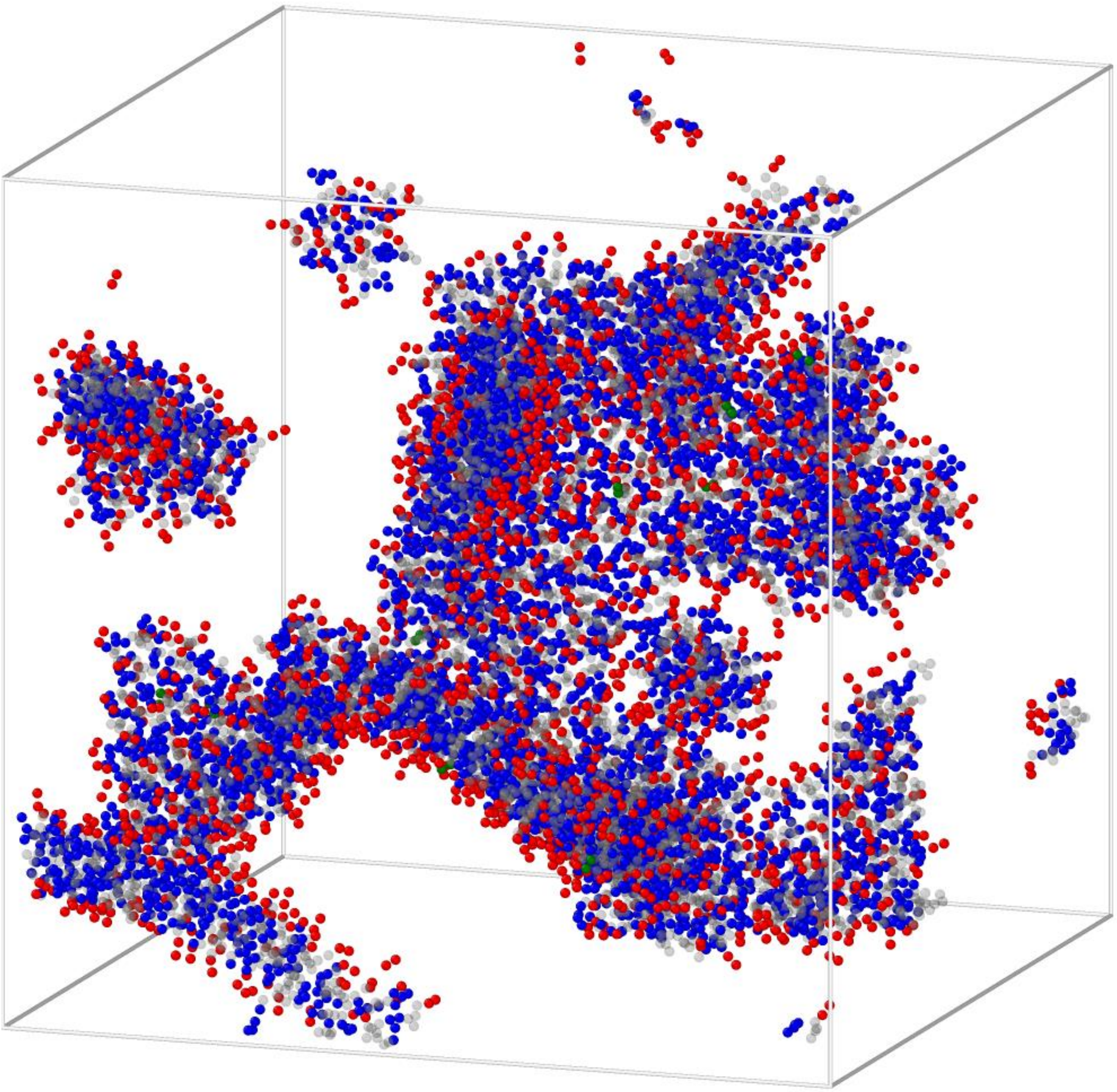

Figure 15. 1200 FFD molecules at pH 4, dark blue beads show protonated F residues (NH3+), red represents deprotonated carboxyl residues (COO-) pink represents protonated carboxyl residues (COOH) while grey represents position 2 F residues. At this pH charge groups are nearly even leading to a near zero net charge and the ability for bilayers to form due to high hydrophilicity paired with charge-charge attraction to stabilize the charged surface.

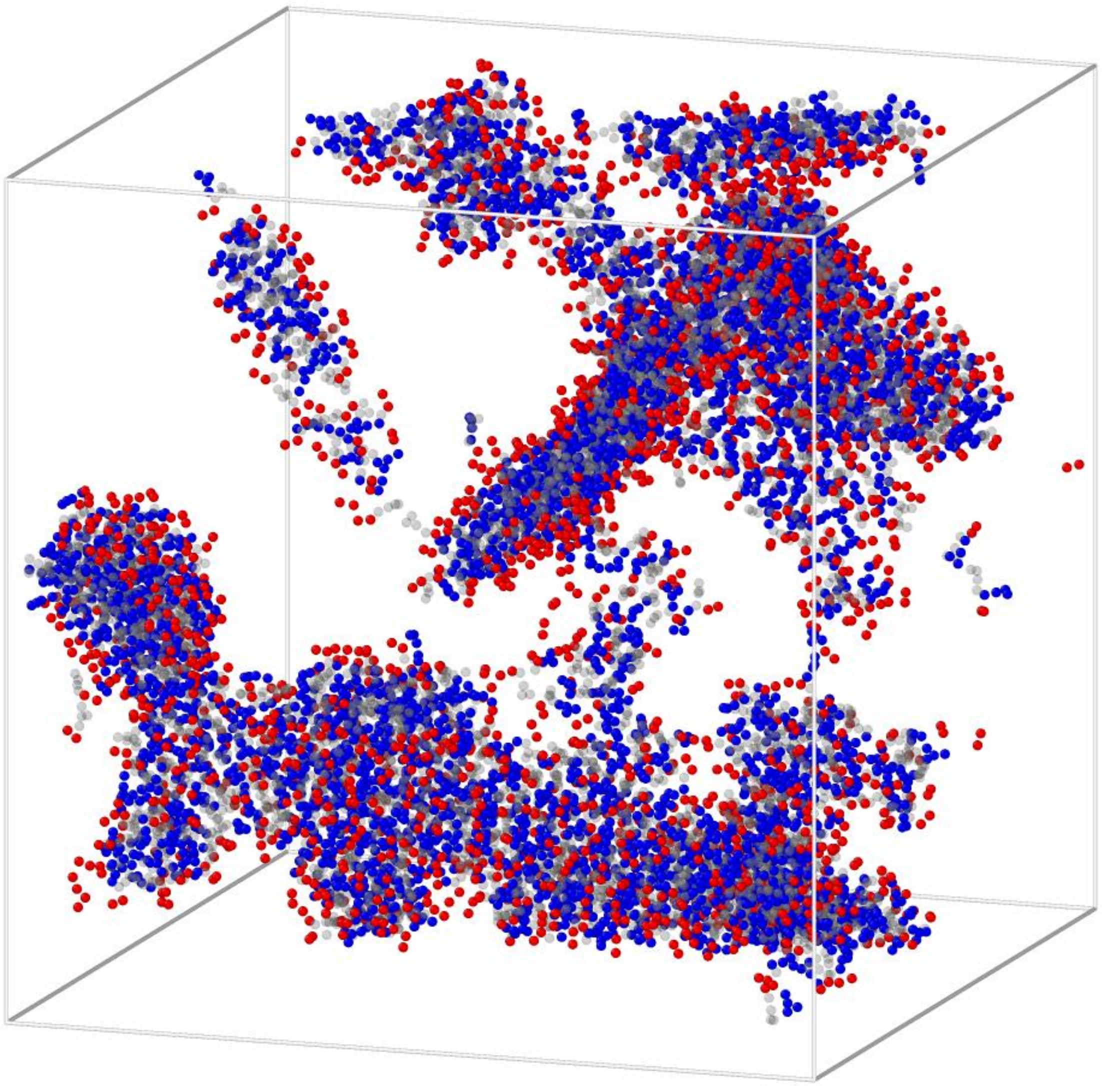

Figure 16. 1200 FFD molecules at pH 5, dark blue beads show protonated F residues (NH3+), red represents deprotonated carboxyl residues (COO-) pink represents protonated carboxyl residues (COOH) while grey represents position 2 F residues. At this pH charge groups are nearly even leading to a near zero net charge and the ability for bilayers to form due to high hydrophilicity paired with charge-charge attraction to stabilize the charged surface.

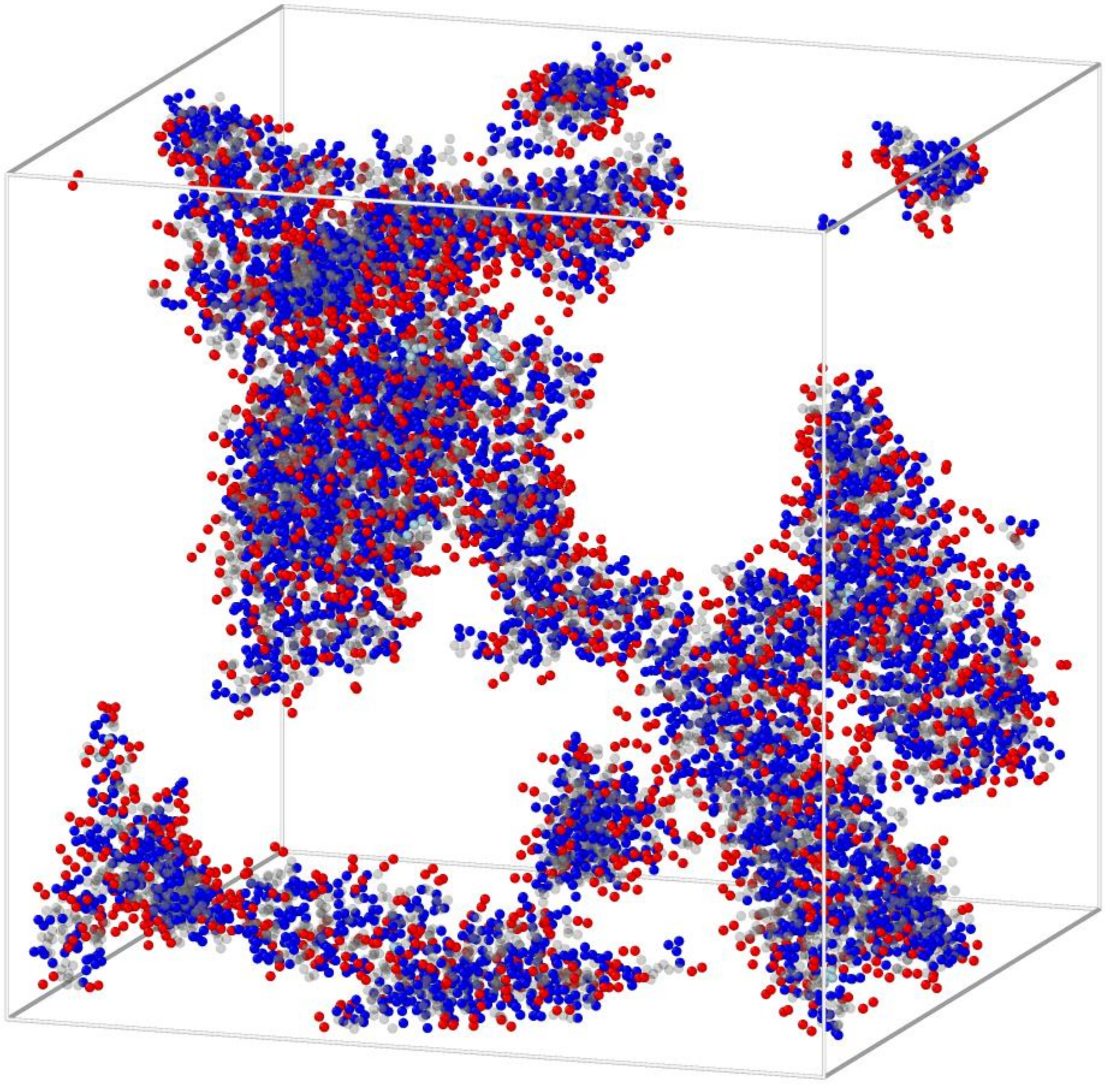

Figure S17. 1200 FFD molecules at pH 6, dark blue beads show protonated F residues (NH3+), red represents deprotonated carboxyl residues (COO-) pink represents protonated carboxyl residues (COOH) while grey represents position 2 F residues. At this pH charge groups are nearly even leading to a near zero net charge and the ability for bilayers to form due to high hydrophilicity paired with charge-charge attraction to stabilize the charged surface.

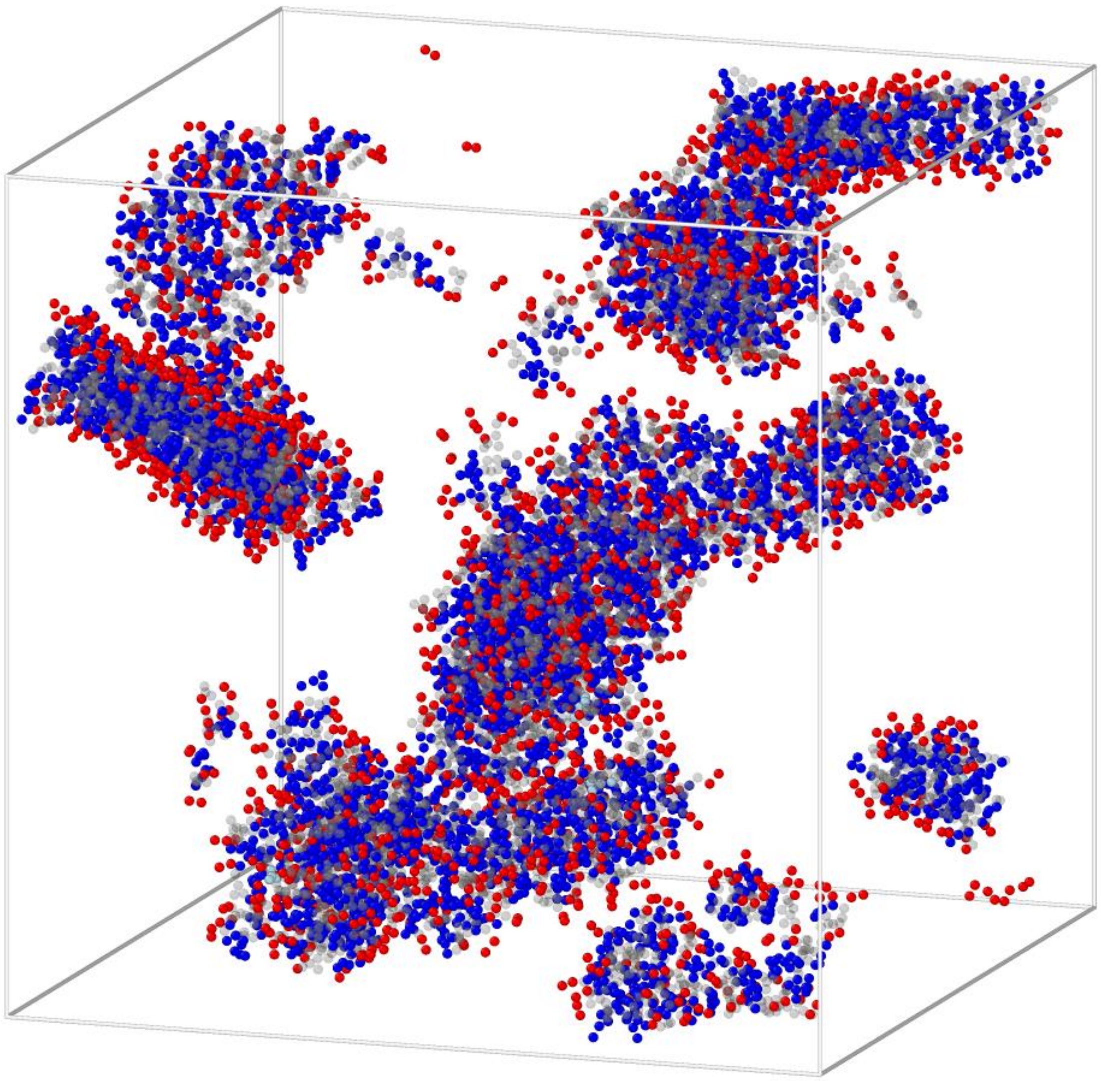

Figure S18. 1200 FFD molecules at pH 7, dark blue beads show protonated F residues (NH3+), red represents deprotonated carboxyl residues (COO-) pink represents protonated carboxyl residues (COOH) while grey represents position 2 F residues. At this pH charge groups are nearly even leading to a near zero net charge and the ability for bilayers to form due to high hydrophilicity paired with charge-charge attraction to stabilize the charged surface.

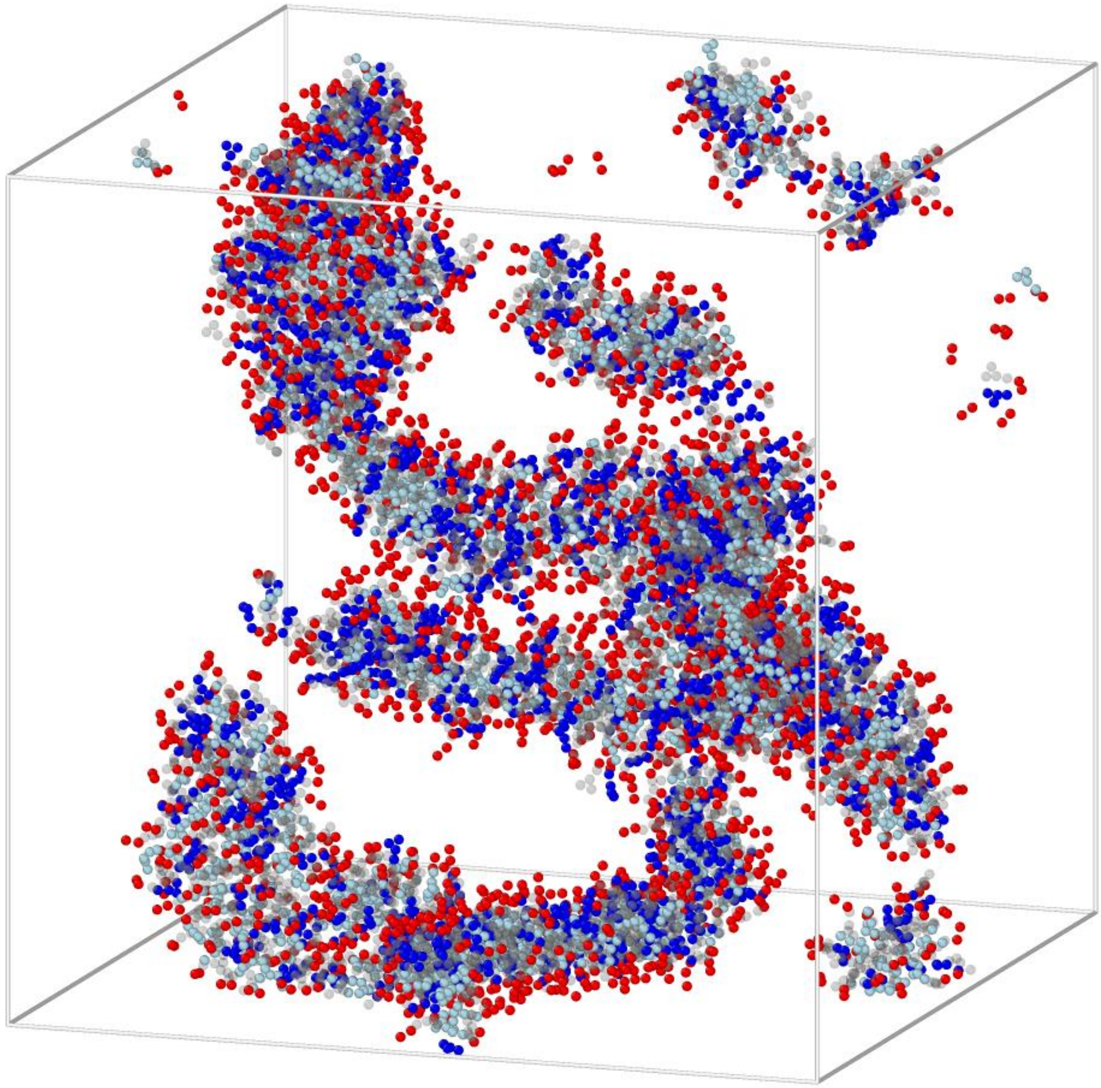

Figure S19. 1200 FFD molecules at pH 8, dark blue beads show protonated F residues (NH3+), red represents deprotonated carboxyl residues (COO-) pink represents protonated carboxyl residues (COOH) while grey represents position 2 F residues. At this pH amine groups are partially protonated leading to a thinning of bilayer structures as negative charges rearrange around the edges.

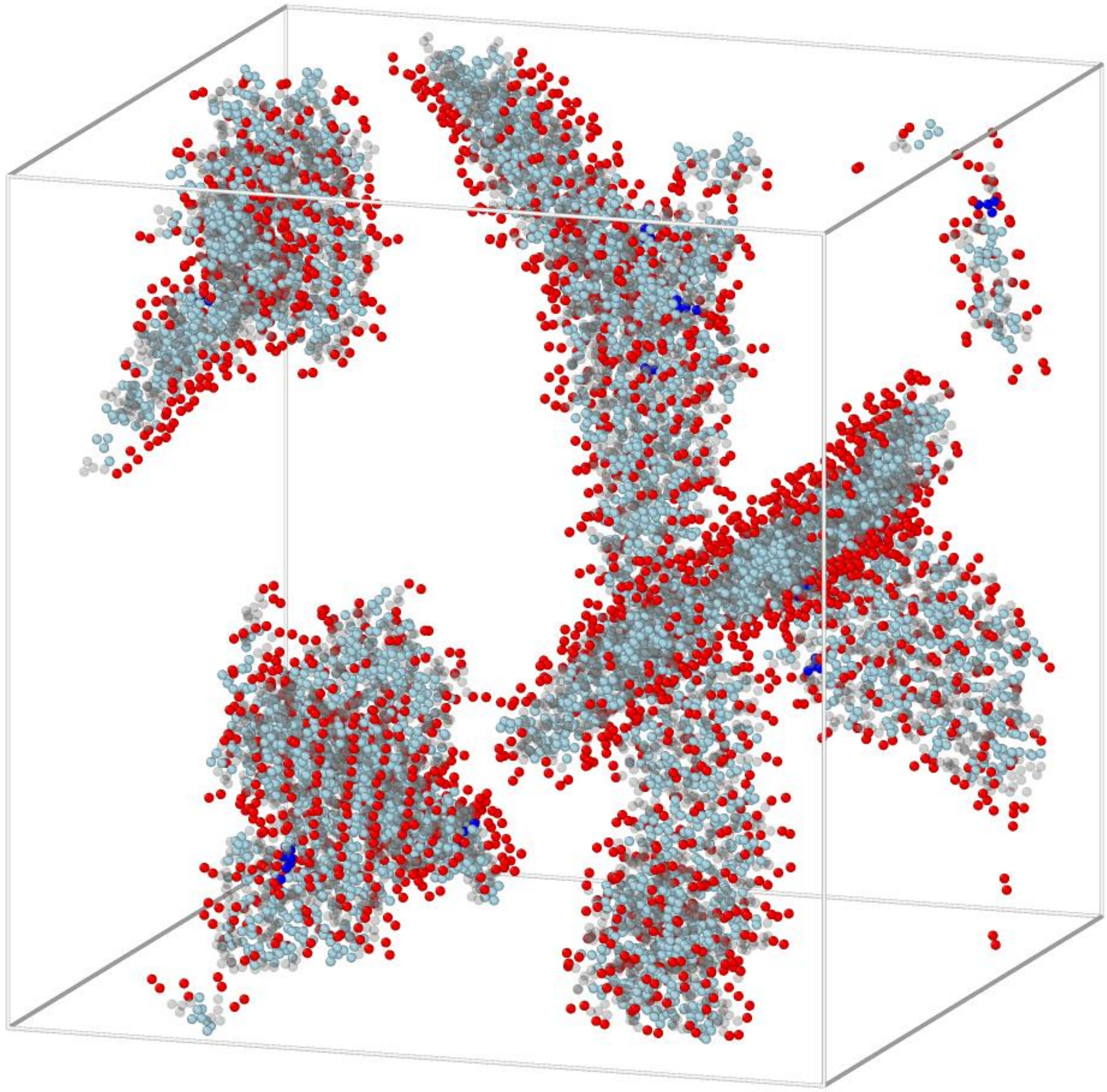

Figure S20. 1200 FFD molecules at pH 9, dark blue beads show protonated F residues (NH3+), red represents deprotonated carboxyl residues (COO-) pink represents protonated carboxyl residues (COOH) while grey represents position 2 F residues. At this pH negative charge groups dominate which leads to charge-charge repulsion forcing negatively charged residues to shift to the edges forming thing nanowires rather than wide bilayers.

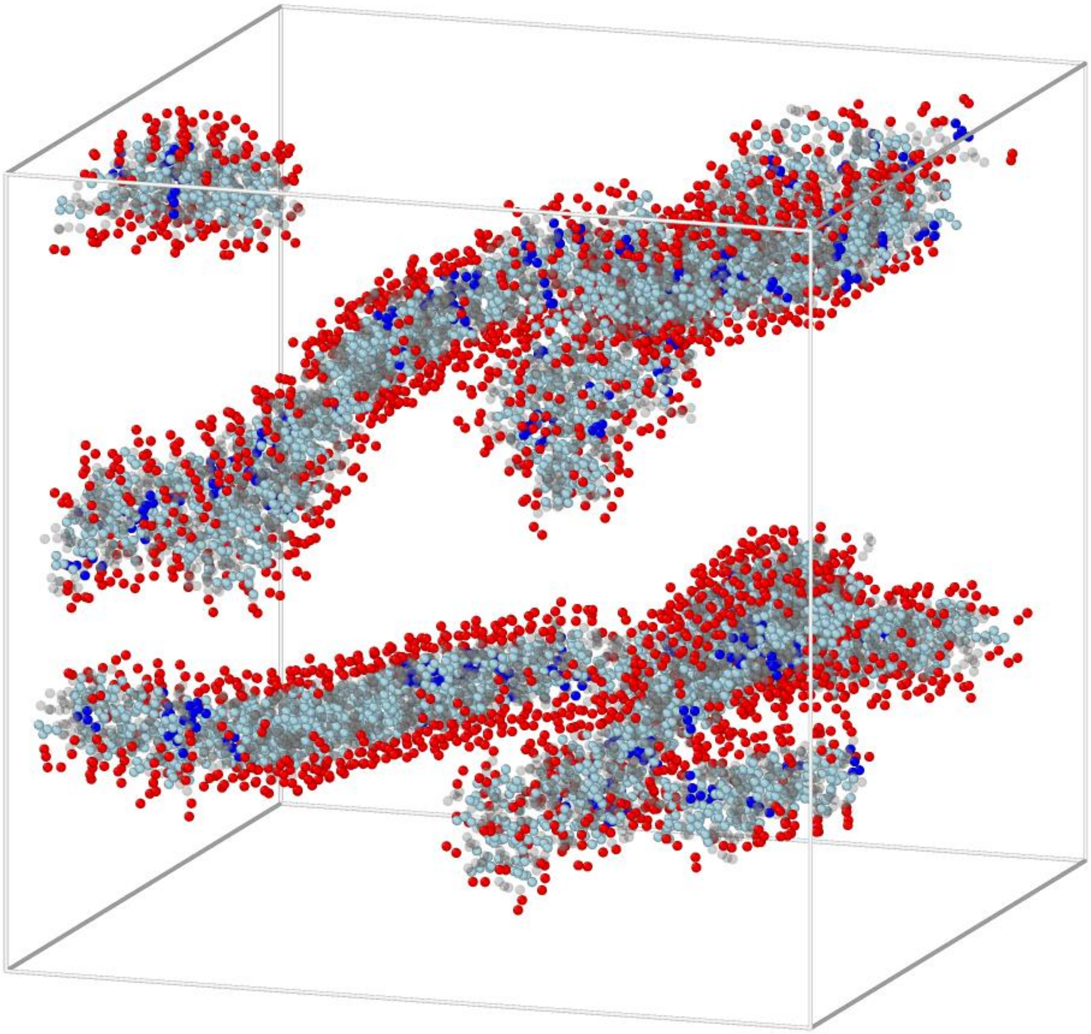

Figure S21. 1200 FFD molecules at pH 10, dark blue beads show protonated F residues (NH3+), red represents deprotonated carboxyl residues (COO-) pink represents protonated carboxyl residues (COOH) while grey represents position 2 F residues. At this pH negative charge groups dominate which leads to charge-charge repulsion forcing negatively charged residues to shift to the edges forming thing nanowires rather than wide bilayers.

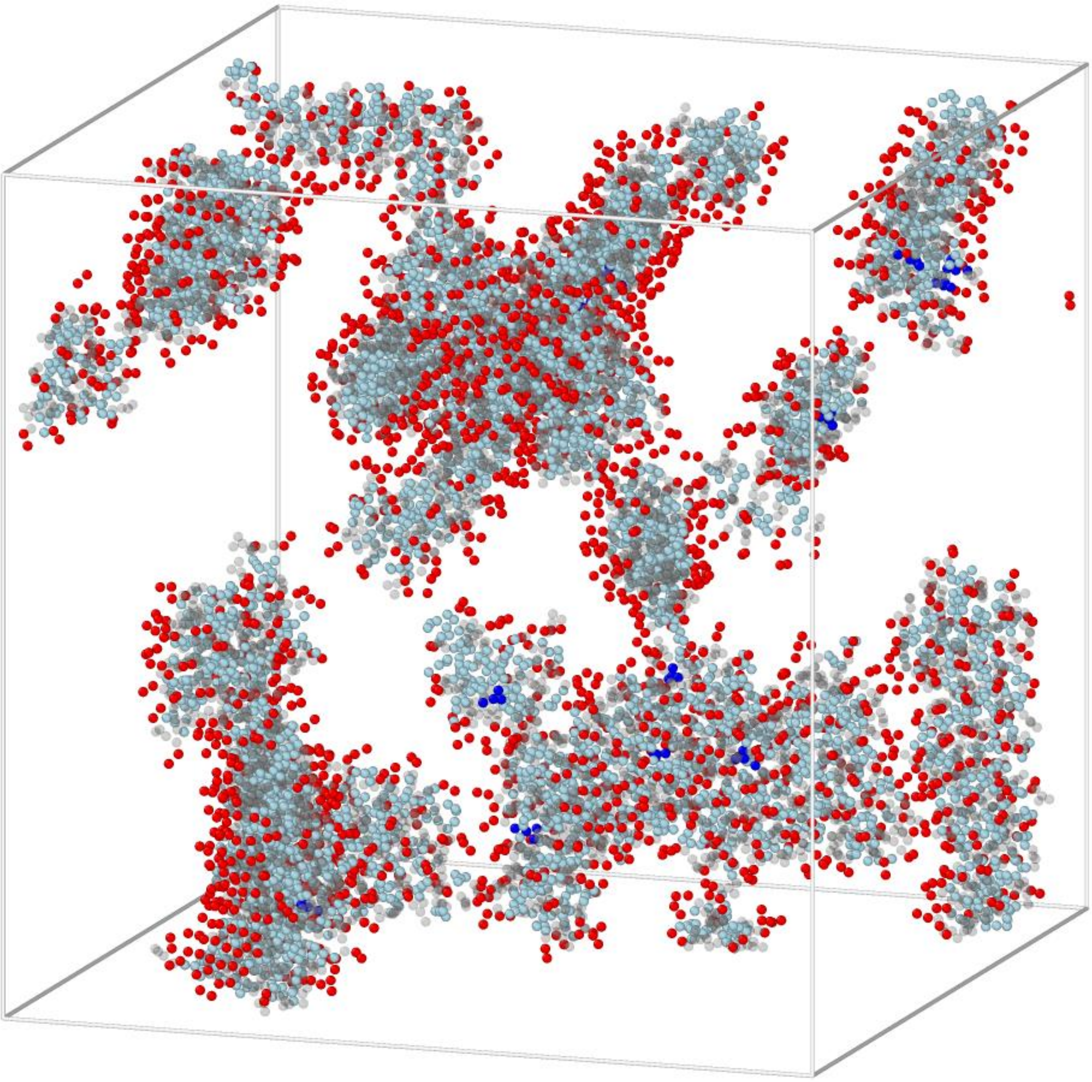

Figure S22. 1200 FFD molecules at pH 11, dark blue beads show protonated F residues (NH_3^+), red represents deprotonated carboxyl residues (COO^-) pink represents protonated carboxyl residues (COOH) while grey represents position 2 F residues. At this pH negative charge groups dominate which leads to charge-charge repulsion forcing negatively charged residues to shift to the edges forming thing nanowires rather than wide bilayers.

3. File Archive

All data underpinning this publication are openly available from the University of Strathclyde KnowledgeBase at https://doi.org/10.15129/f76ac245-0e38-40ca-ac3c-26c000510faf

The authors have included the detailed modified NAMD source core, MD & constant pH outputs files and paths in Table S1. Trajectory files have had frames skipped using MDAnalysis,[4] due to data size limitations.

Table S1. NAMD CpHMD modifications, titratable residue topologies and CpHMD simulation data.

Entry	Description	Data type	File path
1	Modified NAMD source code	Source code.	NAMD_2.14_Source_mod.tar
2	Fmoc CG MARTINI model	NAMD parameters & psfgen topology	Fmoc.zip
3	Oleic acid topology	Psfgen topology	Oleic_acid.top
4	Modified NAMD CpHMD TCL scripts and reference values and parameters for titratable groups	Source code.	CpHMD_scripts_and_reference_values_and_topologies.zip
5	Single oleic acid molecule titration	CpHMD output	1Oleic_CC_VL.zip
6	Oleic acid micelle CpHMD titration with PME electrostatics and constant charge.	CpHMD output	30Oleic_W_CC_VL.zip
7	… PME electrostatics without constant charge	CpHMD output	30Oleic_W_FC_VL.zip
8	… Switch electrostatics without constant charge	CpHMD output	30Oleic_W_CC_noPME_VL.zip
9	… Switch electrostatics without constant charge	CpHMD output	30Oleic_W_FC_noPME_VL.zip
10	Single oleic acid molecule in a POPC bilayer titration	CpHMD output	POPC+1Ole_W_CC.zip
11	Oleic acid titration experimental data	CSV files and python script.	OleicAcidExperimental.zip
12	Single FmocFF titration	CpHMD output	1FmocFF_W_CC.zip
13	10 FmocFF titration	CpHMD output	10FmocFF_W_CC.zip
14	20 FmocFF titration	CpHMD output	20FmocFF_W_CC.zip
15	30 FmocFF titration	CpHMD output	30FmocFF_W_CC.zip
16	40 FmocFF titration	CpHMD output	40FmocFF_W_CC.zip
17	300 FmocFF titration	CpHMD output	300FmocFF_W_CC.zip
18	600 FmocFF titration	CpHMD output	600FmocFF_deprot.zip
19	FmocFF Tube at pH 4 & 9	CpHMD output	FmocFF_Tube_W_CC_2.zip
20	1200 FFD CpHMD	CpHMD output	1200FFD_W_CC.zip

4. References

(1) Sasselli, I. R.; Ulijn, R. V.; Tuttle, T.; Ramos Sasselli, I.; Ulijn, R. V.; Tuttle, T. CHARMM Force Field Parameterization Protocol for Self-Assembling Peptide Amphiphiles: The Fmoc Moiety. *Phys. Chem. Chem. Phys.* **2016**, *18*, 4659–4667.

(2) Yang, Z.; Gu, H.; Fu, D.; Gao, P.; Lam, J. K.; Xu, B. Enzymatic Formation of Supramolecular Hydrogels. *Adv. Mater.* **2004**, *16*, 1440–1444.

(3) Abul-Haija, Y. M.; Roy, S.; Frederix, P. W. J. M.; Javid, N.; Jayawarna, V.; Ulijn, R. V. Biocatalytically Triggered Co-Assembly of Two-Component Core/Shell Nanofibers. *Small* **2014**, *10*, 973–979.

(4) Michaud-Agrawal, N.; Denning, E. J.; Woolf, T. B.; Beckstein, O. MDAnalysis: A Toolkit for the Analysis of Molecular Dynamics Simulations. *J. Comput. Chem.* **2011**, *32*, 2319–2327.
