# Supplementary material for: Constant pH Coarse-Grained Molecular Dynamics with Stochastic Charge Neutralization
Source: J Phys Chem Lett. 2022 Apr 29;13(18):4046–51. doi: 10.1021/acs.jpclett.2c00544 (PMC9109222; doi:10.1021/acs.jpclett.2c00544)
Supplement: Supplementary file 2 — jz2c00544_si_002.pdf [file jz2c00544_si_002.pdf]

jz-2022-00544s.R1

Name: Peer Review Information for "Constant pH Coarse-Grained Molecular Dynamics with Stochastic Charge Neutralization"

First Round of Reviewer Comments

Reviewer: 1

Comments to the Author

The manuscript by Tuttle and co-workers present a new method to perform constant pH coarse-grained molecular dynamics simulations based on Stochastic Charge Neutralization. The method is first presented succinctly, and then most of the manuscript is dedicated to describing the benchmarking simulations using three representative systems: oleic acid micelles and aggregates of Fmoc-FF and FFD peptides. Despite not being a MARTINI/coarse-grained MD expert myself, I consider the method and the reported results to be sound and of wide potential use for the biophysics community. The ability to confidently predict the pKa of complex, aggregating molecules and to alter the ratio of (de)protonated groups at a given pH while correctly modeling the physical phenomena associated to it, is certainly of value for the large-scale simulation of biological systems.

My only criticism is that the manuscript is at times difficult to read. Authors are encouraged to shorten the sometimes very long sentences that make an excessive use of commas. Also, tables and figures could be presented in a clearer way; axes labels should be homogenized (for instance, “deprotonation” and “S” are arbitrarily used). The manuscript legibility would also benefit from moving some of the figures of the Supporting Information to the main text. For instance, I find the formation of peptide nanowires very appealing and non-expert readers would appreciate having a three-dimensional view of the aggregated systems.

Apart from these minor issues, I recommend the manuscript to be published in The Journal of Physical Chemistry Letters.

Reviewer: 2

Comments to the Author

Tuttle and coworkers report the development of a Martini-style coarse-grained constant pH method and validation using three pH-dependent self-assembly systems. The constant pH method is based on the non-equilibrium candidate Monte-Carlo approach developed by the Roux group with modifications to enable net charge neutralization. The latter is accomplished by stochastically altering the charges on water. The methodology and findings are interesting and somewhat novel. However, I have concerns regarding missing citation/comparison to

similar previously developed methods, rigor of the study and suitability for the journal. I think the paper needs to be significantly revised and refined before publication. I agree that the paper presents a methodology of high significance, but it is more suited for J Chem Theory Comput than J Phys Chem Lett which is for succinct papers of high urgency and with potential wide impact. The present form of the paper contains a lot of materials without a clear conclusion. My detailed comments are listed below.

Major concerns:

- The authors claimed that the previous constant pH methods do not account for fluctuating net charge. This is not true. In 2012, the Shen group introduced an approach where some ions are titratable in the system. See Wallace and Shen, J Chem Phys 2012, 137, 184105 https://doi.org/10.1063/1.4766352. They also introduced an approach which makes use of titratable water to offset the fluctuating charges of titratable sites. See Chen, Wallace, Shen, Biophys J 2013, 105, L15. DOI:https://doi.org/10.1016/j.bpj.2013.06.036. This latter approach is similar to what the authors suggest here although detailed implementation differs which has to do with the difference between coarse-grained and atomic force fields.

- Comparison to experiments is rather superficial. The authors compared the calculated pKa's with experiments; however, it is unclear what the exact pKa's are the subjects of comparison. For example, in Table 1, several experimental pKa's are given for different molecules/systems. The authors need to explain in detail which experimental data can be directly compared with their calculations.

- There is no experimental support for the shape of the self-assembled structures.

- Overall, I find that the paper is very loosely written in the sense that many claims are made without going into detailed explanation or citing reference as support. For example, on page 9, "This is due to different structures being able to form (spherical → oblong → fibrous) which decrease the magnitude of the pKa shift and in- creases cooperativity, frames of each self-assembled structure showing structural transition can be found in the Supporting Information, Figure S7-S11. " Why does the morphological change decreases the pKa shift is not explained.

- The figures and tables are poorly prepared. Caption is either incomplete or does not explain the data presented.

Author's Response to Peer Review Comments:

We thank the editor and reviewers for their comments and suggestions and outline in this document the steps we have address the concerns and comments of the reviewers.

To clarify changes that have been made to the original manuscript, red text in the manuscript is in response to Reviewer 1 and blue text is in response to Reviewer 2 – though these do overlap in places.

Editorial Changes

1. *Graphics: Please remove part (a) label from Figure 2. Make appropriate changes to the text of manuscript.*

Removed this insert, thank you for spotting this it is from an earlier version that put together differently.

2. *TOC Graphic: Please resize per journal guidelines (2 in x 2 in) and move to the correct position (on the same page as the abstract).*

We believe the DPI for ACS publications to be 600 (https://pubs.acs.org/page/4authors/submission/graphics_prep.html) and therefore have resubmitted the TOC as 1200x1200 pixels. We have also removed the 'tocentry' function so that the TOC is in the same place as the Microsoft word template version, just above the abstract.

3. *Please remove "Journal of the American Chemical Society" TOC graphic template text (page 18).*

Removed.

4. *Title and Author Lists: Title, author names, and affiliations must match in three places: (1) manuscript file, (2) supporting information, and (3) ACS Paragon Plus.*

This has been done and the changes are described in the table below (green indicates a correction has been made).

	Title	Names	Affiliations
Manuscript	Constant pH Coarse-Grained Molecular Dynamics with Stochastic Charge Neutralization	Alexander van Teijlingen<br>Hamish W. A. Swanson<br>King Hang Aaron Lau<br>Tell Tuttle	Department of Chemistry, University of Strathclyde, 295 Cathedral Street, Glasgow, G1 1XL, UK
Supporting information	Constant pH Coarse-Grained Molecular Dynamics with Stochastic Charge Neutralization	Alexander van Teijlingen<br>Hamish W. A. Swanson<br>King Hang Aaron Lau<br>Tell Tuttle	Department of Chemistry, University of Strathclyde, 295 Cathedral Street, Glasgow, G1 1XL, UK
ACS Paragon Plus	Constant pH Coarse-Grained Molecular Dynamics with Stochastic Charge Neutralization	Alexander van Teijlingen<br>Hamish W. A. Swanson<br>King Hang Aaron Lau<br>Tell Tuttle	University of Strathclyde, Pure and applied chemistry 295 Cathedral St Glasgow, Scotland, UK G1 1XQ

5. *Supporting Information: Please add full header at top of page of the Supporting Information file, which includes: Title, Full Author List, and Author affiliations (exactly as they appear in the manuscript).*

This has now been added.

6. *References: In both the main file and the supporting information, fix the style of all references to use JPCL formatting (check all references carefully). ***JPC Letters reference formatting requires that journal references should contain: () around numbers, author names, article title (titles entirely in title case or entirely in lower case), abbreviated journal title (italicized), year (bolded), volume (italicized), and pages (first-last).*

We have done the following checks and report the results for each citation (green indicates a correction has been made):

<table>
<tr><td>Citation #</td><td>Name(s)</td><td>Title</td><td>Journal</td><td>Year</td><td>Volume</td><td>pages</td></tr>
<tr><td>1</td><td></td><td></td><td>J. Hazard. Mater.</td><td></td><td></td><td></td></tr>
<tr><td>3</td><td></td><td></td><td>Proc. Natl. Acad. Sci. U.S.A.</td><td></td><td></td><td></td></tr>
<tr><td>6</td><td></td><td></td><td></td><td></td><td></td><td>114107</td></tr>
<tr><td>7</td><td></td><td></td><td></td><td></td><td></td><td>024118</td></tr>
<tr><td>8</td><td colspan="6" rowspan="2">These are new citations in response to reviewer 2</td></tr>
<tr><td>9</td></tr>
<tr><td>21</td><td></td><td>The Martini Force Field: Coarse Grained Model for Biomolecular Simulations</td><td></td><td></td><td></td><td></td></tr>
<tr><td>22</td><td></td><td>Acid-Base Catalysis, and Enzymatic Hydrolysis. Part I: Elementary Processes</td><td></td><td></td><td></td><td></td></tr>
<tr><td colspan="7">Supporting Information</td></tr>
<tr><td>1</td><td colspan="6">DOI removed</td></tr>
<tr><td>2</td><td colspan="6">DOI removed</td></tr>
<tr><td>3</td><td colspan="6">DOI removed</td></tr>
<tr><td>4</td><td colspan="6">DOI removed</td></tr>
</table>

Reviewer 1

1. *“My only criticism is that the manuscript is at times difficult to read. Authors are encouraged to shorten the sometimes very long sentences that make an excessive use of commas.”*

Sentences have been reworked & shortened throughout.

2. *Tables and figures could be presented in a clearer way; axes labels should be homogenized (for instance, “deprotonation” and “S” are arbitrarily used)*

We thank the reviewer for pointing out this inconsistency. To address this:

- S is now defined in the first mention of deprotonation.
- Equation 1 has been changed to reflect this.
- Figure 1 has been updated with the y label set to ‘S’ to match the x axis ‘AP’

3. *The manuscript legibility would also benefit from moving some of the figures of the Supporting Information to the main text.*

Due to the letter format guidelines, we are limited to the number of figures allowed in the manuscript. We believe that the main results are of sufficient merit to warrant publication as a letter and while the additional information available in the SI is certainly of interest to those wishing to implement the approach or those studying closely related systems, the inclusion of additional detail in the manuscript would detract from the primary message that is delivered in the current version of the manuscript.

Reviewer 2

1. *The authors claimed that the previous constant pH methods do not account for fluctuating net charge.*

The reviewer is correct in that we had not cited the alternative fluctuating charge methods that were mentioned in their comments. Moreover, they are also correct that our approach is different from these other methods – importantly this difference results from the fact that we are carrying out our simulations within a CG forcefield approach. To highlight this, we have added a comparison to the two alternative methods and highlight the difference between those methods and ours. This additional text is in the fifth paragraph:

"The method we use throughout this work is non-equilibrium molecular dynamics/Monte Carlo (neMD/MC) which benefits from the use of explicit solvent environments while scaling linearly in regard to the number of titratable sites. On top of the original method we have added stochastic charge neutralization which seeks to minimize the number of ions in solution while retaining neutral charge. Previous CpHMD methods developed by Chen & Wallace *et al*.[8,9] have implemented charge neutralization in continuous pHMD (λ-dynamics) to determine all-atom pKa values for six organic molecules and three proteins. These methods reproduced experimental pKa, using residue-ion/water pairs for charge neutralization, more accurately than charge fluctuating methods.[8,9]"

2. *The comparison to experiments in the case of oleic acid is rather superficial.*

In the original Table 1, we compared computed and experimental values for oleic acid. The experimental values from the literature were in different environments, which makes the comparison with the computational results difficult. This is one of the key reasons (in addition to obtaining the Hill coefficients) that we have carried out these experiments ourselves with the results for our own experiments still reported in Table 1. However, we agree with the reviewer that the inclusion of the experimental pKa's obtained under different conditions was not clear in the original table. Therefore, we have removed the entries from Table 1 that aren't directly comparable to our results and instead moved the citation to the footnotes to describe how variable the experimental pKas are under different circumstances.

We have also added more in-text references to the table where we directly compare values to help with the interpretation of the values contained in the table.

3. *There is no experimental support for the shape of the self-assembled structures*

We accept the reviewer's point that, experimentally, the level of detail described in the self-assembled structures is not available. Therefore, we have reworded our discussion of the experimentally observed change in the pKa values to demonstrate how the correlation between the computed pKas and the computed aggregate structures could offer an explanation for this experimentally observed phenomenon. The text following Table 2 has been updated to describe this:

"We rationalise that the higher apparent pK_a (pK_a1) is produced by the different dielectric constant between the surface of the aggregate exposed to water and the monomers buried deep within the aggregate that experience a much lower dielectric environment. The lower apparent pK_a (pK_a2) is due to interacting head groups that proliferate at the aggregate surface at moderate concentration (visualized in the Supporting Information, Figure S5). From the simulations, it is evident that as the nanoaggregate size increases the manoeuvrability of head groups increase which in-part causes the observed re-organization of molecules (spherical →

oblong → fibrous) to maximize head group distance (Supporting Information Figure S6 - S11). This has the effect of decreasing the apparent pK_a and increasing apparent cooperativity due to fewer charge-charge interactions (Figure 2).

4. *Overall, I find that the paper is very loosely written in the sense that many claims are made without going into detailed explanation or citing reference as support (spherical → oblong → fibrous).*

The comparison to structures has been clarified as described in the response to the reviewer's comment #3. Additionally, we have added clarifying text throughout the manuscript to tighten the wording.

5. *The figures and tables are poorly prepared.*

We apologise for this oversight and thank the reviewer for pointing out inconsistencies in the figures. The following changes have now been made:

- Figures 1 & 2 have been changed to remove labels that were accidentally left from previous drafts that no longer serve a clear purpose and do not have descriptions in the caption as well homogenising axis labels to make it clear what they represent.
- Table 1 has been modified from the original which was a very broad survey of the literature which did include molecules and systems that are not directly comparable to our study to a more concise and clear table with the studies removed added as a footnote to provide context for the variable reported pKa values of oleic acid.
- Table 1's title has been entirely reworked to be more concise.
- Table 2's title has been reworded and the note of concentration in MD vs experimental has been moved to a separate footer.
- Figure 2 we have added to the caption to describe the meaning of the purple titration curve.
- Figure 4 has been reworded and parts removed to make it more concise.

jz-2022-00544s.R2

Name: Peer Review Information for "Constant pH Coarse-Grained Molecular Dynamics with Stochastic Charge Neutralization"

Second Round of Reviewer Comments

Reviewer: 1

The authors addressed most of the issues raised by the reviewers. The manuscript can be accepted for publication in The Journal of Physical Chemistry Letters, but grammar has to be seriously revised, either at this or at the proofs stage. For instance:

The sentence (page 2, lines 34-37) "Partly due to its conflict with the first constraint where the number of particles are initially and persistently defined." hardly makes sense, since it comes from a longer sentence that has been split.

Also, the sentences "In validating this methodology as well as exploring use-cases three self-assembling systems of increasing complexity and with existing experimentally quantified pKa changes or pH dependent phase changes [were --missing--] investigated. The first being the commonplace oleic acid micelle titration which serves as a general evaluation of CpHMD methods." Sentences cannot just be split without rewording, please have a deeper look: "The first studied system was the ..." would read better.

In the sentence "with the likelihood of deprotonation (S) at a given pH is evaluated against the Metropolis criteria", either "with" should be "where", or "is evaluated" should be "being evaluated".

"...were rendered by the software package..." should read "...were rendered with the software package..."

Please format Table 1 in a more visually attractive way, with lines or boxes separating the values from the headers, etc.

Reviewer: 2

The authors addressed my comments and improved the quality of their manuscript.
